# Methylammonium Lead Iodide across Physical Space: Phase Boundaries and Structural Collapse

**DOI:** 10.1021/acs.jpclett.4c03336

**Published:** 2024-12-23

**Authors:** Pelayo Marin-Villa, Mattia Gaboardi, Boby Joseph, Frederico Alabarse, Jeff Armstrong, Kacper Drużbicki, Felix Fernandez-Alonso

**Affiliations:** † Materials Physics Center, CSIC-UPV/EHU, Paseo de Manuel Lardizabal, 5, 20018 Donostia - San Sebastian, Spain; ‡ C.S.G.I. and Chemistry Department, University of Pavia, Viale Taramelli 16, 27100 Pavia, Italy; ¶ Elettra Sincrotrone Trieste S.C.p.A., Strada Statale 14, 163.5, Basovizza, 34149 Trieste, Italy; § ISIS Neutron and Muon Facility, Rutherford Appleton Laboratory, Didcot OX11 0QX, United Kingdom; ∥ Polish Academy of Sciences, Centre of Molecular and Macromolecular Studies, Sienkiewicza 112, 90-363 Lodz, Poland; ⊥ Donostia International Physics Center (DIPC), Paseo de Manuel Lardizabal 4, 20018 Donostia - San Sebastian, Spain; # Ikerbasque, Basque Foundation for Science, Plaza Euskadi 5, 48009 Bilbao, Spain

## Abstract

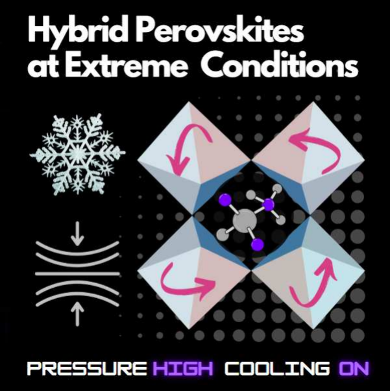

Hybrid perovskites exhibit complex structures and phase behavior under different thermodynamic conditions and chemical environments, the understanding of which continues to be pivotally important for tailoring their properties toward improved operational stability. To this end, we present for the first time a comprehensive neutron and synchrotron diffraction investigation over the pressure–temperature phase diagram of the paradigmatic hybrid organic–inorganic perovskite methylammonium lead iodide (MAPbI_3_). This ambitious experimental campaign down to cryogenic temperatures and tens of kilobars was supported by extensive *ab initio* molecular dynamics simulations validated by the experimental data, to track the structural evolution of MAPbI_3_ under external physical stimuli at the atomic and molecular levels. These combined efforts enable us to identify the mechanisms underpinning structural phase transitions, including those exhibiting negative thermal expansion across the boundary between the cation-ordered low-temperature phase and the dynamically disordered high-pressure cubic phase. Our results bring to the fore how pronounced octahedral distortions at high pressures ultimately drive the structural collapse and amorphization of this material.

Hybrid organic–inorganic perovskites (HOIPs) have attracted substantial interest and attention owing to their remarkable optoelectronic properties, making them excellent candidates for applications in photovoltaics, light-emitting diodes, and photodetectors.^1^ Despite this potential, HOIPs still exhibit intrinsic and extrinsic instabilities that hamper their commercial utilization.^2,3^ Although encapsulation and surface passivation can minimize environmental sensitivity to moisture, UV radiation or oxidation, the most critical issues are still deeply rooted in the intrinsic and hard-to-control fragility of the perovskite framework, leading to ion migration and eventual decomposition.^4,5^ These obstacles are further exacerbated when integrating the perovskite with interfaces and charge-transport layers.^2^ Materials design, new synthetic routes or interfacial engineering, to name a few, have emerged as possible routes to circumvent these limitations. They all rely on the exceptional tunability of HOIPs and their exceedingly low elastic stiffness,^6^ which can be explored by introducing chemical pressure–e.g., through cation engineering–or by controlling residual strains via ion doping.^7,8^ In this context, understanding the flexibility and associated (de)stabilization pathways of soft perovskite frameworks under external stimuli has received increasing attention. In particular, physical pressure can provide new insights into the stability of thermodynamic phases and structural distortions without the need to effect chemical modifications to the material. These considerations also pave the way for the development of more effective strategies overcoming current operational limitations.

Generally speaking, three-dimensional HOIPs conform to a canonical and ubiquitous ABX_3_ structure, where an organic cation occupies the lattice A–site within the cuboctahedral cavity formed by the soft, formally anionic metal-halide framework BX_3_. The inorganic environment provided by the BX_3_ sublattice comprises corner-shared octahedra that can be easily distorted. In turn, the softness of these structures results in the emergence of rather-complex phase behavior, and a coherent understanding of the latter still remains beyond our reach. This situation continues to be the case for methylammonium lead iodide, MAPbI_3_, and its P–T phase diagram is summarized in Figure 1.

**Figure 1:**
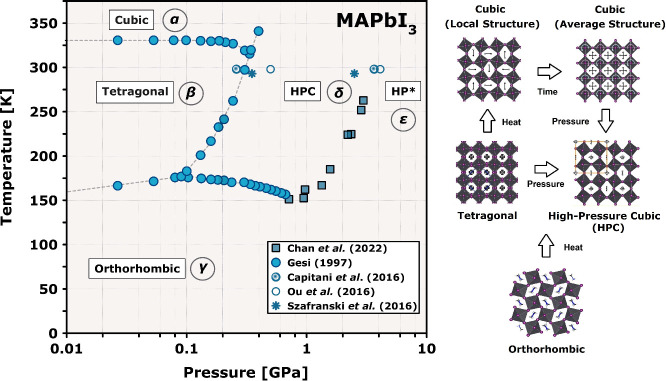
P–T phase diagram of MAPbI_3_, as inferred from dielectric/infrared spectroscopy^9,10^ and X-ray diffraction.^11−13^ Greek letters are used to label the thermodynamically stable phases reported in the literature. Selected projections of the structures of these phases are depicted at the right. The average, nonequilibrium structure of the cubic phase is displayed along with an instantaneous local structure obtained from total-scattering synchrotron diffraction,^14^ to provide a visual comparison with the average structures of the tetragonal and high-pressure cubic phases.^13^

At ambient pressure, crystallography reveals three perovskite phases in the case of MAPbI_3_: a cubic phase above 330 K; a tetragonal phase over the range 160–330 K; and an orthorhombic phase below 160 K.^15^ We designate these phases as α, β and γ, respectively. Following the seminal work of Gesi in the kbar regime,^10^ a number of subsequent studies have accessed higher pressures–see Table S1 for a summary of work to date. From these results, we know that there are at least two compressed states: a high-pressure cubic phase and a postperovskite amorphous phase–hereafter denoted as δ and ϵ, respectively. The δ phase has been previously studied only at room temperature,^13,16^ thus key structural information on its thermal evolution either below or above ambient conditions is still missing. Similarly, very little is known about the local structure and stability regime of the ϵ phase. To fill these gaps in our current understanding of MAPbI_3_, we report the first extensive survey of the P–T phase diagram and the associated regimes of stability of MAPbI_3_ using a combination of neutron and synchrotron X-ray diffraction–henceforth ND and XRD, respectively. These experimental data are then used to guide a detailed mapping of these hitherto unexplored regions of physical space via the use of *Ab Initio* Molecular Dynamics, AIMD, simulations, to explore the properties of MAPbI_3_ at the atomic and molecular levels.

Figure 2 shows representative ND and XRD patterns for MAPbI_3_ at pressures of up to tens of kbar. Using the space-group assignments of Szafrański and Katrusiak,^13^ diffraction patterns could be fitted adequately, with average weighted-profile *R*-factors of 2 and 7%, and relative goodness-of-fit between 0.85 and 1.53. As starting point, we confirmed that the resulting volumetric parameters at ambient pressure agree with those reported earlier by Lehmann et al.^17^ and summarized in Figure S1. Diffraction patterns along the 2 kbar isobar indicate an essentially continuous character associated with the α → β transition, in agreement with previous works.^18^ On the other hand, both β → δ and δ → γ transformations exhibit a rather discontinuous character. Our structural assessment of the phase boundaries along the 2 kbar isobar agrees with the dielectric-spectroscopy results of Gesi.^10^ Moreover, the X-ray diffraction patterns along the T = 295 K isotherm displayed in Figure 2b confirm the occurrence of a β → δ phase transformation at 3 kbar–see also Table S1. At room temperature, we also observed another δ → ϵ transition in the pressure range 2.5–3.0 kbar, accompanied by a significant reduction of the unit-cell volume by ca. 5%, a result which is in line with previous reports.^13^ The compressed regime progressively evolves toward a highly disordered structure,^19^ as evinced by a substantial broadening of Bragg features and a noticeable increase of the diffuse background, indicating the loss of long-range order. Using the bespoke experimental setup described in Section S2.1 and depicted in Figure S2, we gained access to previously unexplored high-pressure and low-temperature regimes in MAPbI_3_, down to ca. 120 K and 20 kbar. The diffraction patterns collected under these conditions revealed that Im3̅ symmetry was always preserved upon cooling at high pressure No signatures of the highly disordered ϵ phase were found.

**Figure 2:**
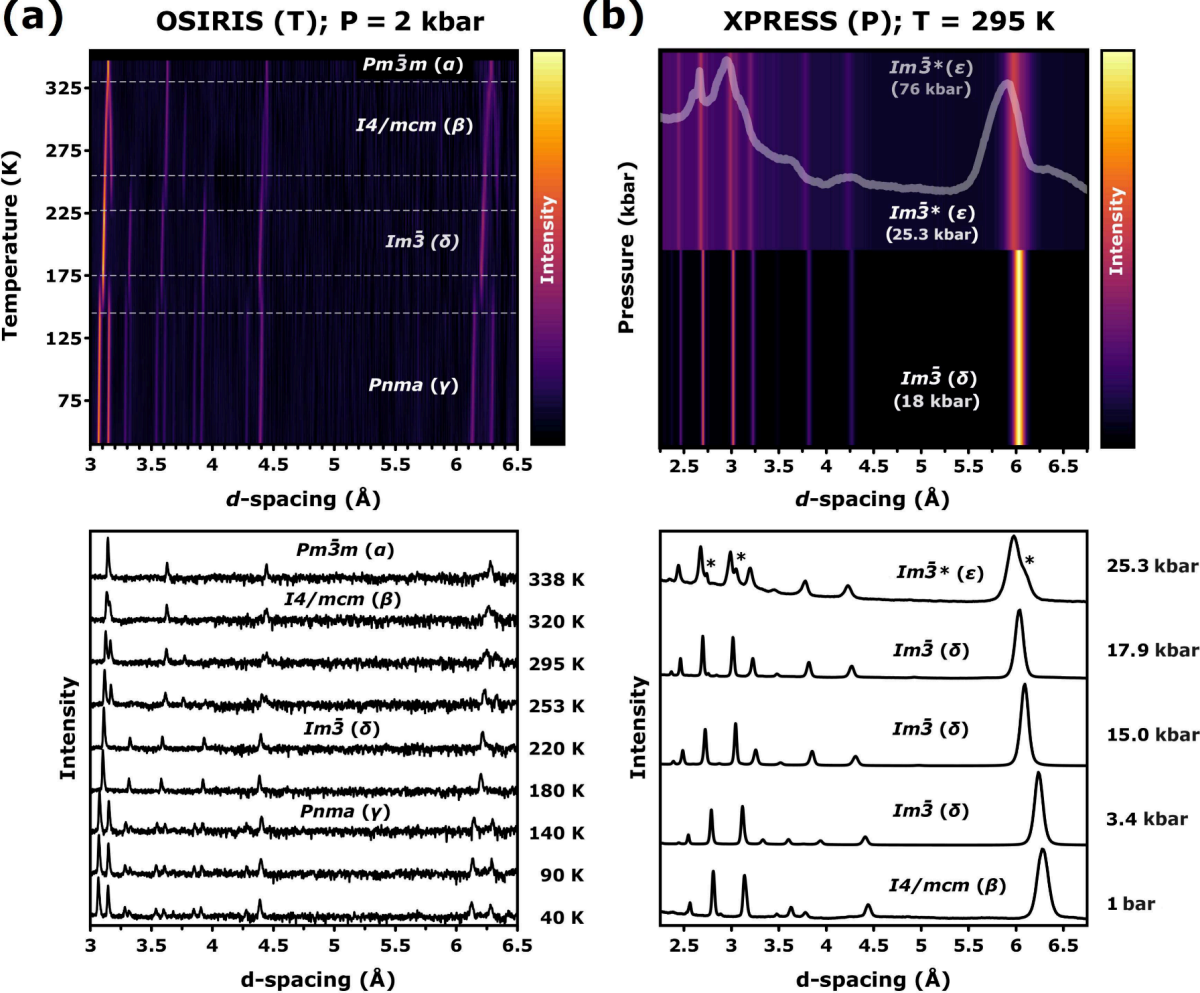
Representative (a) ND and (b) XRD patterns for a powder specimen of MAPbI_3_. In line with the regimes highlighted in Figure 1, the ND patterns obtained on OSIRIS are displayed as a function of temperature along the 2 kbar isobar. The XRD data from Xpress correspond to the ambient-temperature isotherm. Raw diffraction patterns are presented in the bottom panels, while the interpolated color maps in the top panels highlight the evolution of the diffraction patterns across each phase transition. The horizontal dashed lines at the top of panel a highlight the transition points. The diffraction pattern for a highly pressurized ϵ phase (*Im*3̅*, 76 kbar) is presented as an inset in panel b. The appearance of new diffraction features across the δ → ϵ transition is marked with asterisks in panel b.

Figure 3 reports the evolution of the Formula-Unit Density (FUD) across the P–T diagram of MAPbI_3_. This quantity allows for a model-independent assessment of these data irrespective of the above-adopted space-group structural assignments. In addition, it enables direct comparison with computational predictions. In this context, we note that the search for robust structural models of MAPbI_3_ has proven a formidable task from a computational point of view. Besides the well-known perovskite phases, Flores-Rivas et al.^20^ found two nonperovskite structures at lower energies (−15 meV/FU) using the SCAN functional. Further work combining Monte Carlo and machine learning techniques suggested that a double-delta structure was thermodynamically preferred below 200 K.^21^ Our analysis of the experimental data at temperatures as low as 10 K, however, could not provide further evidence supporting the presence of a nonperovskite phase in our samples. Building upon this and our previous works on structural models of the γ phase,^22,23^ the current *in-silico* predictions take as input a series of possible structural models of MAPbI_3_, namely, *Pnma*, *P*1, and *Cmcm* (see Figure S3). Their primary differences relate to the orientations of the methylammonium cations, particularly in terms of the hydrogen-bonding environment between organic and inorganic sublattices. The methylammonium cations of the *Pnma* model show an end-to-end ordering along an evenly hydrogen-bonded configuration, as shown in Figure S3. On the other hand, the cations of the *P*1 and *Cmcm* models lose this end-to-end ordering, leading to a weakening of the hydrogen bonds with the inorganic sublattice. Computationally, a systematic screening of P–T space requires the (rather challenging) use of spatial scales containing of the order of a thousand atoms, as well as semilocal functionals such as PBEsol.^24^ Within these approximations, AIMD at the PBEsol level is able to capture structural subtleties like the transient distorted domains of the α-phase, and the microscopic mechanisms behind phase transformations.^25^ These results are in line with experimental data and time-independent DFT.^14,26^ Access to these scales and functional approximations using the workflow outlined in Figure S4 also provides a means of benchmarking the latter by direct comparison with experimental data.^27^ Within this framework, the computational results tend to lift phase boundaries by ca. 3 kbar and 50–100 K relative to observation. As shown in Figure S9, a spontaneous γ → β transition is seen at ca. 250 K at ambient pressure, a value to be compared to ca. 160 K observed in the laboratory. These mismatches in transition temperatures are linked to the use of semilocal DFT along with the classical sampling of time inherent to AIMD.^28,29^ Notwithstanding, the computational prescription adopted herein seems to provide a reasonably accurate depiction of the phase behavior of MAPbI_3_ for comparison with experimental input. In terms of the FUD landscape across the P–T plane, the results of the AIMD simulations presented in Figure 3b also reveal differences between the *Pnma* and *P*1 models. In support of this assertion, Section S3 of the SI describes an extended set of computational results. At 1 bar, differences between these two structural models occur mainly in the low-temperature regime, as reflected by considerably different Vibrational Density Of States – VDoSs, see Figure S5. Figure S7 shows that the emergence of a tetragonal phase while crossing the γ → β boundary from below is retained in both models, resulting in the same vibrational band structure and competing thermodynamic stability–see also Figures S5 and S6.

**Figure 3:**
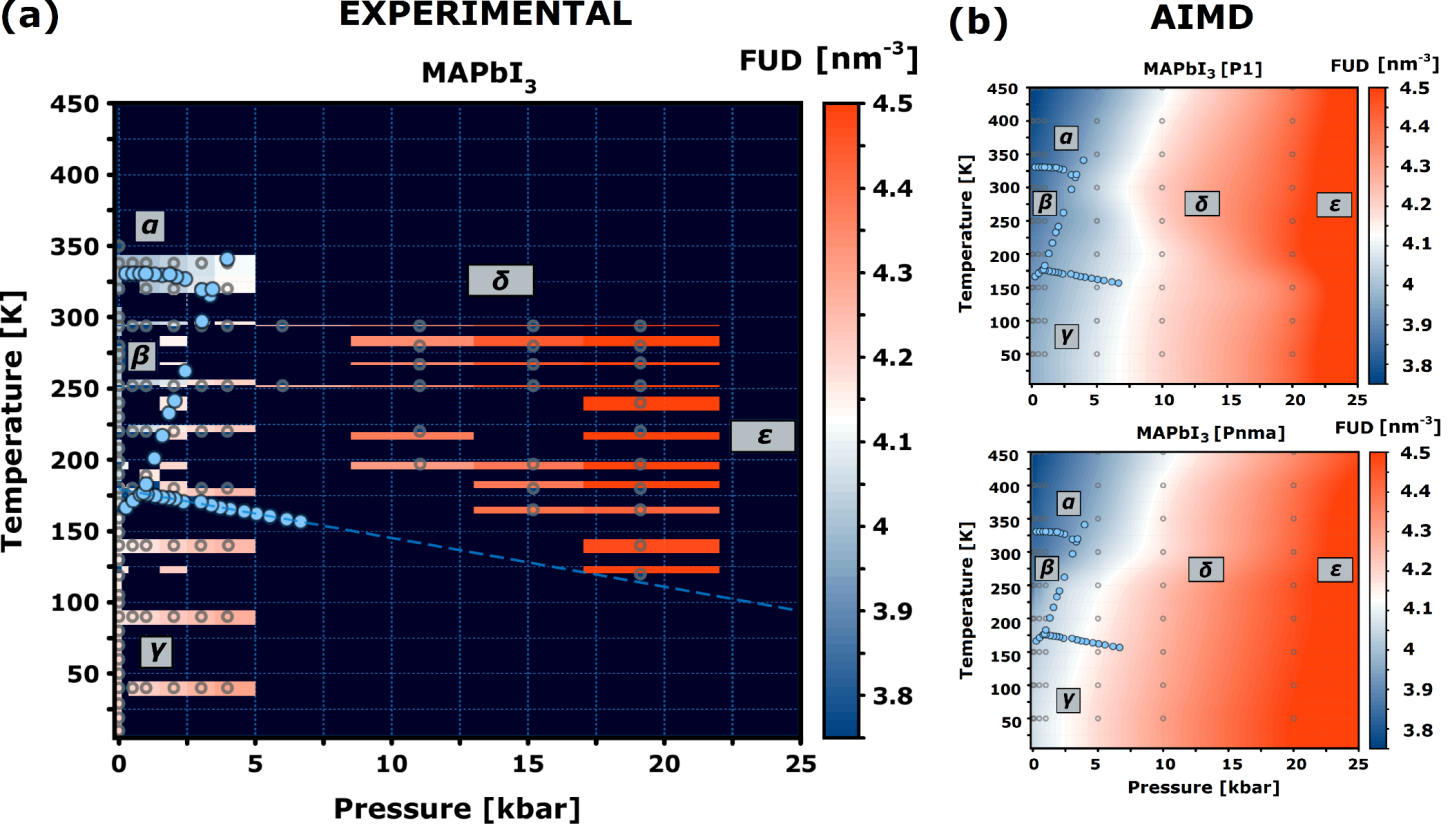
(a) Reconstructed P–T phase diagram of MAPbI_3_ up to 25 kbar, obtained from the ND and XRD data. (b) Corresponding phase diagrams for the *P*1 and *Pnma* structural models, obtained with AIMD simulations in the NPT ensemble. The heat maps in panels a and b give the corresponding FUDs, as defined in the text. The two heat maps presented in panel b were spatially interpolated from the full set of data up to 100 kbar. Thermodynamically stable phases are indicated by the same Greek letters as those defined above. The empty points in panels a and b correspond to the specific P–T points explored in either the experiments or the calculations. Filled blue points were taken from the dielectric spectroscopy studies by Gesi.^10^

The behavior of MAPbI_3_ with temperature at ambient pressure has already been the subject of previous studies.^25,28−33^ Armed with our new experimental and computational data presented above, we can now attempt to address for the first time the mechanism driving the transition between the cation-ordered low-temperature γ phase and the high-pressure δ phase. As shown by the isobars reported in Figure S7, the *Pnma* model can evolve into the β structure at ambient pressure, yet visual inspection of Figures S8 and S11 shows that it does not form a representative structure of the δ phase at higher pressures. On these grounds, it is not possible to descend continuously through these specific pathways–see Figure S9.^34^ Instead, octahedral tiltings in both *P*1 and *Cmcm* models allow for transformation into the high-pressure structure with maximal in-phase tilting, in line with the characteristic *a*^+^*b*^+^*c*^+^ scheme of the δ phase which has been experimentally observed – ψ0ψ in Alexandrov notation, as shown in Figure S10.^13^ Although no signatures of the highly disordered ϵ phase have been detected at low temperatures, further insights into the structure of this phase may be inferred from the theoretical simulations. All of our calculations indicate the disappearance of the well-defined corner-shared perovskite structure beyond 20–25 kbar, with a considerable compression of the resulting postperovskite phase. A clear structural collapse and formation of a nonorthorhombic, disordered phase is found at 50 kbar. This result is insensitive to the choice of the initial model–see Figure S12. From an analysis of the pair-correlation functions presented in Figure S13, this transformation is reflected by a substantial shortening of Pb–Pb distances by about 0.5 Å and by the emergence of a secondary I–I correlation at ca. 5 Å.

Figure 4 provides further quantitative insights into the experimentally determined FUD as a function of both temperature and pressure. Panels 4a and 4b show six isobars over the temperature range 40–350 K. In the low-compression regime preceding the low-temperature triple-point at 175 K and 1 kbar, we observe a continuous cell expansion, with a noticeable drop of the FUD at ca. 150 K marking the γ → β transition–see Figure 4a. The analysis shown in Table S2 of section S4 links the sudden reduction of the FUD to a volume increase of roughly 4.7 Å^3^, and a concomitant increase of the entropy in 30 J/kg·K. Figure 4c contains further information regarding the thermal evolution of the FUD. The zero-temperature FUD ρ_0_ shown in this figure increases monotonically with pressure, whereas α exhibits the opposite trend. In both cases, the effects are far more pronounced above 10 kbar. On the high-temperature end, α undergoes an order-of-magnitude decrease, while the behavior at temperatures below the above-mentioned triple point is radically different, exhibiting a thermal compressibility roughly 20 times larger than what is observed at the higher temperatures. Such behavior defines MAPbI_3_ as a super expandable material in the regime of low temperatures and pressures.

**Figure 4:**
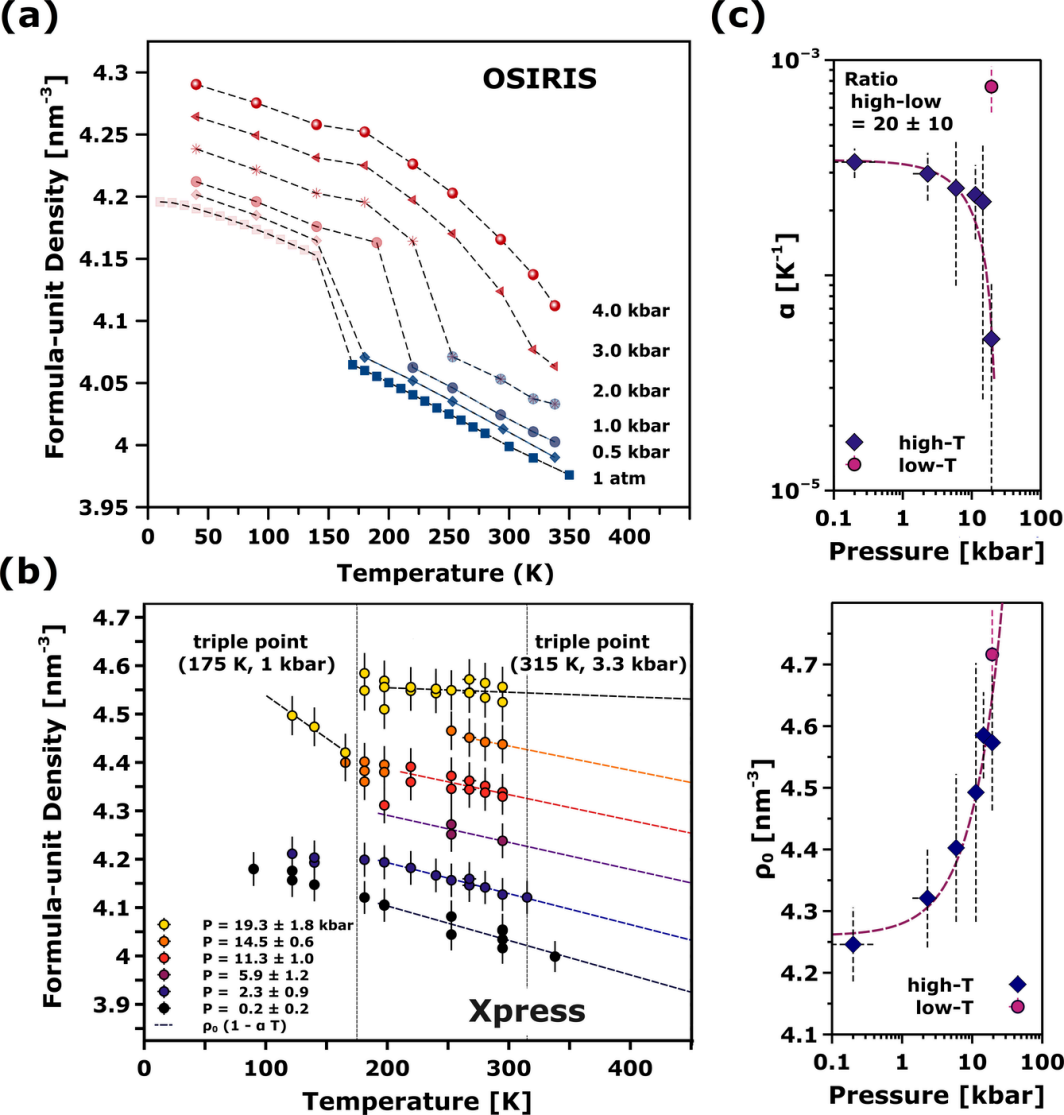
Temperature dependence of the FUD along isobars (a, ND) up to 4 kbar and (b, XRD) in the range of 0.2–20 kbar. The abrupt density increase in panel b corresponds to the transition to the high-pressure cubic phase at low temperature and high pressure. (c) Resulting parameters describing the temperature and pressure dependence of the FUD using the equation ρ = ρ_0_(1 – α*T*).

As soon as the applied pressure drives the perovskite to the δ phase, we observe a slight but measurable cell contraction accompanying the γ → δ transition in the vicinity of the above-mentioned triple point, corresponding to the onset of Negative Thermal Expansion–hereafter NTE. This effect is far more pronounced in the high-pressure regime probed with XRD (see Figure 4b). The AIMD simulations also enable an independent appraisal of the *Pnma*, *P*1 and *Cmcm* models introduced earlier. Figure 5 illustrates that the *Cmcm* and *P*1 models of the γ phase can qualitatively reproduce the NTE observed in the experiments. Both models possess a reduced head-to-tail ordering of the organic moieties with respect to the stiffer *Pnma* model, which cannot describe this behavior as a function of temperature. This result highlights the relevance of cation alignment in dictating the structural properties of HOIPs, which is directly related to the hydrogen-bonding environment. In this sense, it is shown that models with a weakened hydrogen-bonding environment can provide insights not only of the spectroscopic response but also of the structural properties of MAPbI_3_. We also note that NTE is unique for the case of the γ → δ transformation. In contrast, Figure 4a shows a rather-abrupt cell expansion of roughly 5.6 Å^3^ when MAPbI_3_ undergoes the δ → β transformation–see also Table S2.

**Figure 5:**
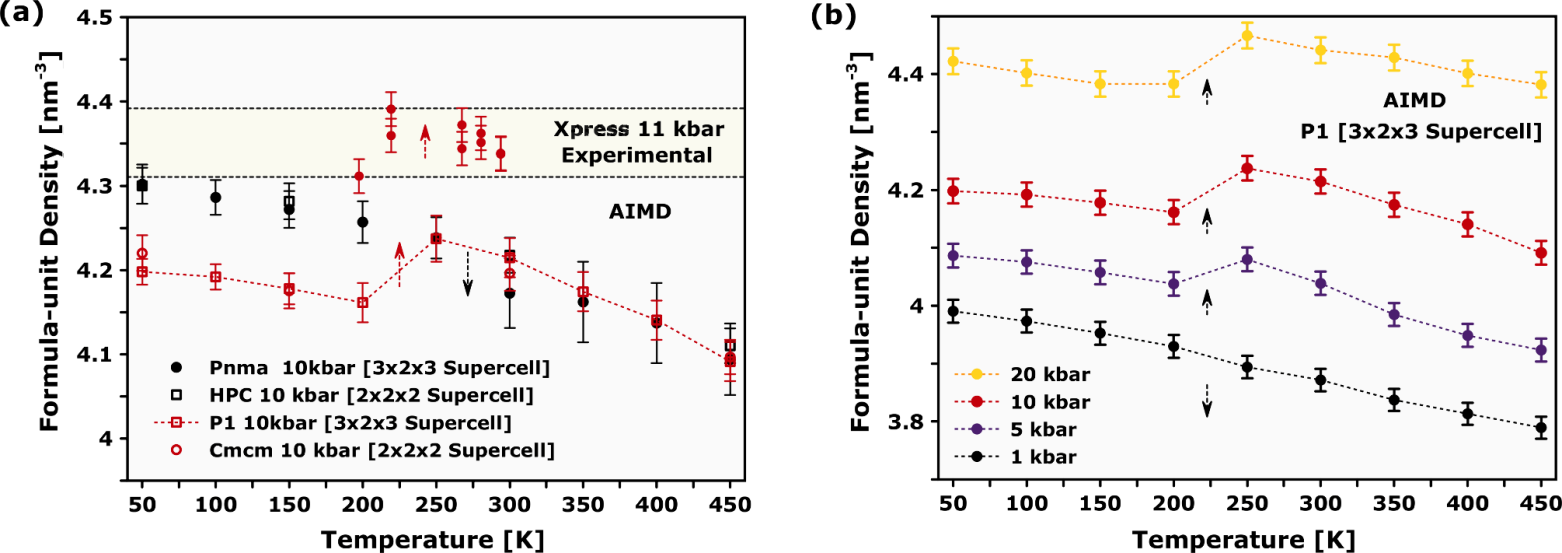
(a) Temperature evolution of the FUD from synchrotron X-ray diffraction (Xpress) and NPT AIMD simulations at 10 kbar (see the figure legend for details). (b) FUD as a function of temperature and pressure (1–20 kbar), obtained with the *P*1 model.

In summary, we have carried out the first systematic exploration of the P–T plane of MAPbI_3_ using radiation-scattering techniques in tandem with AIMD simulations. These efforts have enabled us to describe in unprecedented detail the behavior of MAPbI_3_ across phase boundaries and on its way to structural collapse. On the basis of these results, we can rule out the possibility of a postperovskite ϵ structure in the low-temperature, high-pressure end, as simulations indicate a loss of the perovskite structure irrespective to the initial model. Our FUD analysis highlights the emergence of NTE across the hitherto-unexplored discontinuous transition at low temperatures and moderate pressures. The *P*1 and *Cmcm* models introduced in our earlier works^22,23^ can reproduce the observed trends in the experimental data, and provide a consistent microscopic description of the evolution of the corner-shared perovskite framework under physical stimuli. Accordingly, the set of computational results highlight the importance of the role played by the alignment of the organic cation on structural properties, not only of the γ phase but also on its evolution with pressure. To the best of our knowledge, this is the first time that a detailed picture of the structural evolution up to the onset of collapse has been put together for MAPbI_3_. Likewise, we anticipate that further studies on the effect of pressure on the stochastic motions of the methylammonium cation across the phase diagram will provide additional insights not amenable to scrutiny using diffraction techniques.

## Experimental and Computational Details

Experiments were performed using the same MAPbI_3_ sample as in previous works.^22,23,35,36^ The high-pressure diffraction experiments using synchrotron radiation and neutrons were performed on Xpress (Elettra Synchrotron, Italy) and OSIRIS (ISIS Neutron and Muon Source, Rutherford Appleton Laboratory, United Kingdom), respectively.^37,38^ The AIMD simulations were performed with the CP2K code,^39^ using the PBEsol functional.^24^ The simulations covered the temperature and pressure range of 50–450 K and 1 atm–100 kbar, respectively, up to 50 ps on an ensemble of up to 1000 atoms. The outputs were processed with TRAVIS and OVITO.^40,41^ Further experimental and computational details are provided in Section S2 of the SI.

## Supporting Information Available

Phase transitions in MAPbI_3_, experimental and computational details, additional computational results, and quantitative analysis of volumetric properties (PDF)
