# Supplementary material for: Methylammonium Lead Iodide across Physical Space: Phase Boundaries and Structural Collapse
Source: J Phys Chem Lett. 2024 Dec 23;16(1):184–90. doi: 10.1021/acs.jpclett.4c03336 (PMC11726799; doi:10.1021/acs.jpclett.4c03336)
Supplement: Supplementary file 1 — jz4c03336_si_001.pdf [file jz4c03336_si_001.pdf]

# Supplementary Information for Publication: Methylammonium Lead Iodide across Physical Space – Phase Boundaries and Structural Collapse

Pelayo Marin-Villa,[†] Mattia Gaboardi,[†,‡] Boby Joseph,[¶] Frederico Alabarse,[¶] Jeff Armstrong,[§] Kacper Drużbicki,[*,‖] and Felix Fernandez-Alonso[*,†,⊥,#]

†*Materials Physics Center, CSIC-UPV/EHU, Paseo de Manuel Lardizabal, 5, 20018 Donostia - San Sebastian, Spain*

‡*C.S.G.I. & Chemistry Department, University of Pavia, Viale Taramelli, 16, 27100, Pavia, Italy*

¶*Elettra Sincrotrone Trieste S.C.P.A., Strada Statale 14, km 163.5, Basovizza, 34149 Trieste, Italy*

§*ISIS Neutron and Muon Facility, Rutherford Appleton Laboratory, Didcot, OX11 0QX, United Kingdom*

‖*Polish Academy of Sciences, Centre of Molecular and Macromolecular Studies, Sienkiewicza 112, 90-363 Lodz, Poland*

⊥*Donostia International Physics Center (DIPC), Paseo de Manuel Lardizabal 4, 20018 Donostia - San Sebastian, Spain*

#*IKERBASQUE, Basque Foundation for Science, Plaza Euskadi 5, 48009 Bilbao, Spain*

E-mail: kacper.druzbicki@cbmm.lodz.pl; felix.fernandez@ehu.eus

# Contents:

## S1. Phase Transitions in $MAPbI_3$

## S2. Experimental and Computational Details

### S2.1. High-pressure Diffraction

### S2.2. Computational Details

## S3. Additional Computational Results

### S3.1. Vibrational Properties

### S3.2. Evolution of the Perovskite Structure Across the P–T Space

## S4. Quantitative Analysis of the Volumetric Properties

# S1. Phase Transitions in $MAPbI_3$

$MAPbI_3$ reveals complex phase behavior across pressure and temperature axes whose understanding remains elusive to date. As such, models derived from the average structure and space-group assignments inferred from crystallographic methods have been a matter of ongoing debate. For instance, the total-scattering experiments at ambient pressure of Beecher *et al.* have revealed the existence of fluctuating symmetry-broken domains of size $1 - 3$ nm caused by the combined effects of octahedral tilting and off-centering of both MA and Pb cations, quite distinct to the cubic-structure models derived from standard diffraction experiments – see the insets in Fig. 1 in the main text.[1] Other works also highlight the importance of intrinsic twinning and the emergence of ferroelectric/ferroelastic properties in the $\beta$ phase of $MAPbI_3$.[2–6] In addition, the low-temperature $\gamma$ regime exhibits substantial crystal twinning,[7] considerable diffuse scattering,[8] as well as reveals inconsistencies of the commonly accepted structural model when confronted with thermophysical and spectroscopic data.[9–12]

Table S1: Pressure-induced phase transitions in $MAPbI_3$ at ambient temperature reported in the literature. The Greek labels correspond to those defined in the main text. ND stands for Neutron Diffraction

| **Phase Sequence** [$\beta$ / $\delta$ / $\epsilon$] | **Method** | **Reference** |
|---|---|---|
| $I4/mcm$ / $Imm2$ (7 kbar) / (41 kbar) | Powder XRD | Ou *et al.*[13] |
| $I4/mcm$ / $Imm2$ (0.26 kbar) | Powder XRD | Capitani *et al.*[14] |
| $Fmmm$ / $Im\overline{3}$ (3 kbar) | Powder XRD | Jaffe *et al.*[15] |
| $Fmmm$ / $Im\overline{3}$ (7 kbar) | Single-crystal XRD | Jaffe *et al.*[15] |
| $I4/mcm$ / $Im\overline{3}$ (4 kbar) / $Immm$ (27 kbar) | Powder XRD | Jiang *et al.*[16] |
| $I4/mcm$ / $Imm2$ (4 kbar) | Single-crystal XRD | Kong *et al.*[17] |
| $I4/mcm$ / $Im\overline{3}$ (45 kbar) / $Im\overline{3}$ (25 kbar) | Single-crystal XRD | Szafrański *et al.*[18] |
| $I4/mcm$ / $Imm2$ (3 kbar) / $Immm$ (27 kbar) | Powder ND / Powder XRD | Kong *et al.*[19] |

Similar considerations apply to studies at higher pressures. Table S1 provides a compilation of high-pressure diffraction experiments on $MAPbI_3$ reported to date in the literature. At first glance, it highlights some ambiguities in space-group assignments across the P–T phase diagram. At ambient temperature, X-ray Diffraction –XRD– of $MAPbI_3$ powder indicate a $\beta \rightarrow \delta$ transition doubling the number of formula-units per unit cell, from $N = 4$ to

$N = 8$. The results from Jaffe *et al.*,[15] suggest that the transition is from the orthorhombic hettotype of $I4/mcm$, $Fmmm$ with $N = 8$, although this assignment was later ruled out in favor of the parent tetragonal phase.[18] Other authors assign the $\delta$ phase to orthorhombic symmetry of *Imm2* type, yet this assignment has been put into question.[18]

# S2. Experimental and Computational Details

## S2.1. High-pressure Diffraction Experiments

High-pressure diffraction experiments were performed using the same sample of $MAPbI_3$ as in our previous works (CAS No 69507-98-8, purity > 99 %; Xi'an Polymer Light Technologies) .[10,11] The ND experiment was carried out using the OSIRIS time-of-flight (backscattering) spectrometer at the ISIS Neutron and Muon Facility (STFC, Didcot, UK).[20–24] An aluminum sachet containing 8 g of powder was wrapped in a cylinder and placed inside a Ti-6Al-4V-alloy, TAV6, gas-pressure cell (⌀7 mm) operating in the pressure range 0–5 kbar. The pressure was controlled externally using a gas intensifier connected to the cell in order to maintain it constant while changing the temperature. A $NaCAlF_6$ reference sample was measured at room temperature in a vanadium cell and employed subsequently to refine both instrument and profile parameters. The cell was screwed on a centerstick and vertically inserted into a Closed Cycle Refrigerator (CCR). Diffraction patterns were collected at different isobars (*i.e.*, 1 bar and 0.5, 1, 2, 3, and 4 kbar) in the temperature range of 10–350 K. Raw time-of-flight data were acquired using the $d_4$, $d_5$, and PG002 detectors (in a nearly backscattering geometry) and subsequently reduced to $d$-spacing utilizing Mantid suite for neutron & muon analysis,[25] roughly covering the 2.5–7.5 Å range. Data from different detectors were merged in Matlab, while Le Bail analyses to extract the cell parameters were carried out using the GSAS-ii suite.[26] Acquisition times were selected to map the phase diagram by recording ND data for over fifty distinct points across the P-T plane. Figure S1 shows that our results of the ambient-pressure isobar are in excellent agreement with the

synchrotron data reported earlier by Lehmann *et al.*[27]

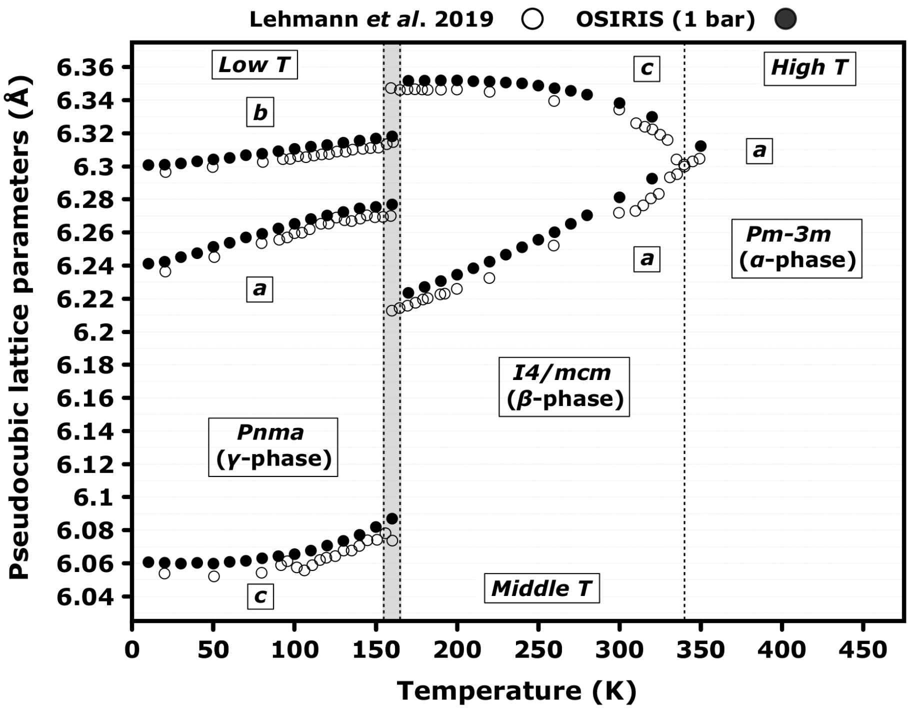


Figure S1: Temperature dependence of the pseudocubic lattice parameters of $MAPbI_3$ at ambient pressure. The filled points are derived from the NPD experiment on OSIRIS (present work), whereas the empty points are due to synchrotron X-ray diffraction, according to Lehmann and co-workers.[27] Both experiments refer to the hydrogenous powder specimens.

High-pressure XRD measurements were carried out on the Xpress beamline[28] at the Elettra synchrotron light source (Trieste, Italy), employing a monochromatized 25 keV beam. A small amount of powder was carefully grinded in an agate mortar and loaded in an 11.5 mm Tozer-Xray Diamond Anvil Cell (DAC; Almax Easylab; with a maximum loading pressure of 200 kbar), using a 5:1 mixture of iso-propanol:*n*-pentane as pressure transmitting medium. The pressure was controlled using the ruby fluorescence method, where a few ruby chips were placed inside within the gasket area. In some scans, a fraction of NaCl was also added to cross-check the pressure values from diffraction. Owing to the small size of the DAC, a purpose-built cooling system was developed by coupling an Oxford System Cryojet with an in-house developed cell cylinder specifically designed to prevent ice formation on the DAC and to control the temperature in the range 120–300 K (see Fig. S2).

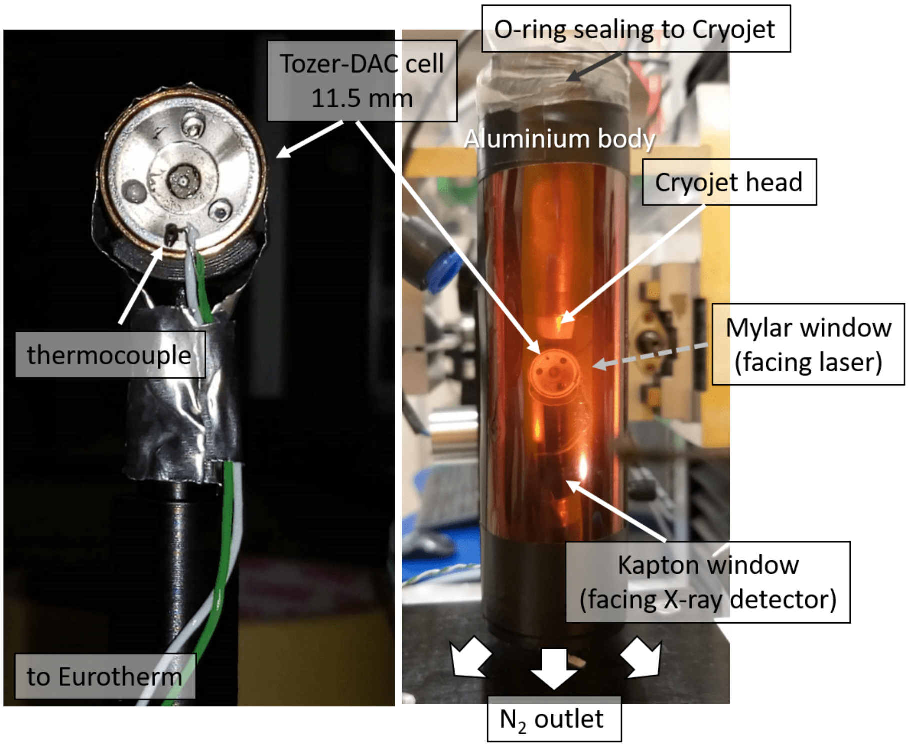


Figure S2: The Tozer-DAC cell (left) along with the cooling cell (right) preventing the moisture condensation.

The experiment was carried out in different runs, each starting by applying a different pressure at room temperature, followed by cooling and stabilization at various temperatures. For each temperature, various fluorescence scans were performed until stabilization, followed by a high-statistic collection and pressure determination by fitting the $R_1$ and $R_2$ peaks of ruby with pseudo-Voigt functions, using calibration method by Datchi *et al.*[29] The high-pressure cell was realigned at each change of P–T and the powder patterns were recorded using a 2D MAR-345 image plate detector (calibrated at room temperature using $CeO_2$ from NIST). The integration of the 2D powder patterns (after masking for spurious features) and subsequent data refinement were performed using GSAS-ii.[26] Due to the non-linear change in pressure with temperature, the data were regrouped in sets of isobar values, and errors were propagated accordingly.

## S2.2. Computational Details

The time-evolved simulations of large supercell structures of $MAPbI_3$ were performed across its P–T phase diagram using the solid-state formulation of Density Functional Theory (DFT) as implemented in the CP2K code.[30,31] The convergence criteria were chosen to ensure a good compromise between the accuracy and performance of expensive *An Initio* Molecular Dynamics (AIMD) simulations and match the numerical quality of the earlier-reported AIMD campaigns for $MAPbI_3$.[32–35] An efficient direct energy functional minimization method, using the Orbital Transformation (OT) scheme, was employed for the Self-Consistent Field (SCF) calculations with the target accuracy of $5\times10^{-7}$ a.u. The ionic forces were calculated using the semi-local PBEsol functional.[36] As a part of broader experimental and computational efforts of our team,[10,37] the selection of the PBEsol functional was motivated by *i.)* its widespread use by the perovskite community, *ii.)* its reduced computational cost, *iii.)* and its ability to avoid hindering the cation dynamics revealed by the pairwise dispersion-corrected DFT+D schemes.[38–40] The hybrid GPW method that combines Gaussian basis sets with auxiliary Plane Waves (PWs) was used to calculate the electronic structure. The atom-centered double-zeta quality polarized basis sets (DZVP-MOLOPT) and Goedecker-Teter-Hutter (GTH) pseudopotentials optimized for PBEsol were used in combination with the set of PWs with an energy cutoff of 300 Ry.

Following our previous works on the low-temperature structure of $MAPbI_3$,[37] the simulations considered alternative models of the $\gamma$ phase as the starting point, namely the *Pnma*, *P*1, and *Cmcm* (each one confirmed as the local minimum on the potential energy landscape of $MAPbI_3$). All three models feature a slightly different local structure around the organic cation along with the associated hydrogen-bonding geometry (details in Fig. S3 and ref.[37]). Particularly, alternative models possess reduced head-to-tail ordering of the methylammonium cations with respect to the *Pnma* phase, which is considered as the energetically lowest-energy configuration with the strongest hydrogen-bonding interactions.[37] For the sake of comparison, the simulations also considered the room-temperature model of the $\delta$ phase

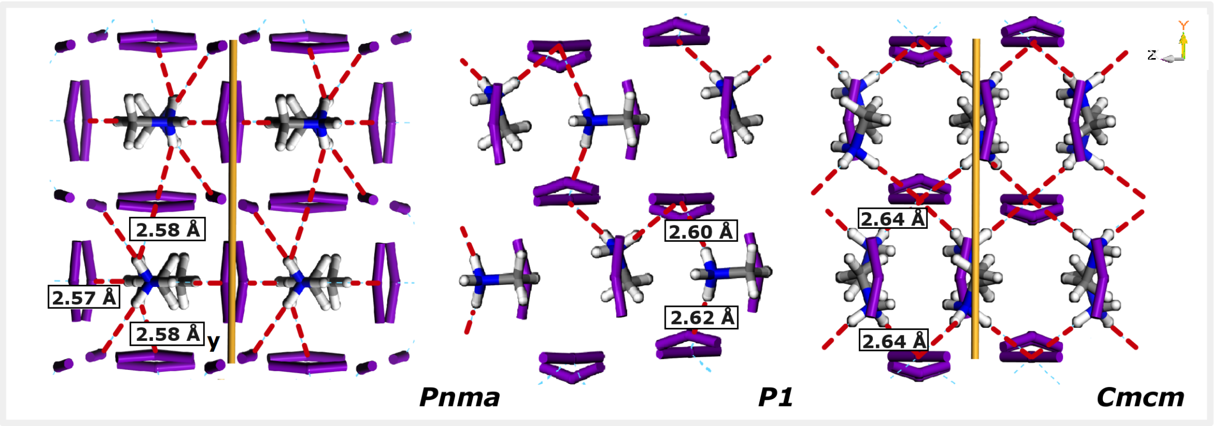


Figure S3: Different cation alignment and related hydrogen-bond geometry (dashed red curves) in three alternative models of the cation-ordered phase of $MAPbI_3$ (*Pnma*, *P*1, and *Cmcm*) according to ref.[37] For ease of visualization, the conventional unit cells are projected along the *y*-axis (marked as a gold line). The purple sticks denote the lead-iodine bonding, whereas the Pb atoms are omitted from the display.

model by Szafrański and Katrusiak according to single-crystal XRD at 23.4 kbar – hereafter, *HPC*.[18]

Fig. S4 provides the computational workflow adopted in the present work. The calculations were performed using extended supercells, accommodating for 72 (*Pnma* and *P*1; unit cell multiplied by 3×2×3) and 64 (*Cmcm* and *HPC*; unit cell multiplied by 2×2×2) formula units, respectively. First, simulations were performed in the isothermal-isobaric ensemble (NPT), with all the atoms explicitly represented with their real masses. The temperature was controlled by a Nose-Hoover thermostat using three chains, whereas Martyna's barostat controlled the pressure.[41] The time constant for both the thermostat and the barostat was set to 50 fs. For both *Pnma* and *P*1 models, NPT runs were initiated at 50 K at a given pressure. Subsequent trajectories were continued by gradually increasing the temperature across the 50–450 K range and performing the simulations for at least 50 ps at given thermodynamic conditions. For the *Cmcm* and the *HPC* models, the calculations were performed without introducing any thermal history, i.e., by directly compressing/decompressing the structure at a given pressure and temperature. No constraints on the cell shape were imposed, allowing for volume fluctuations by changing the edges and angles of the supercell.

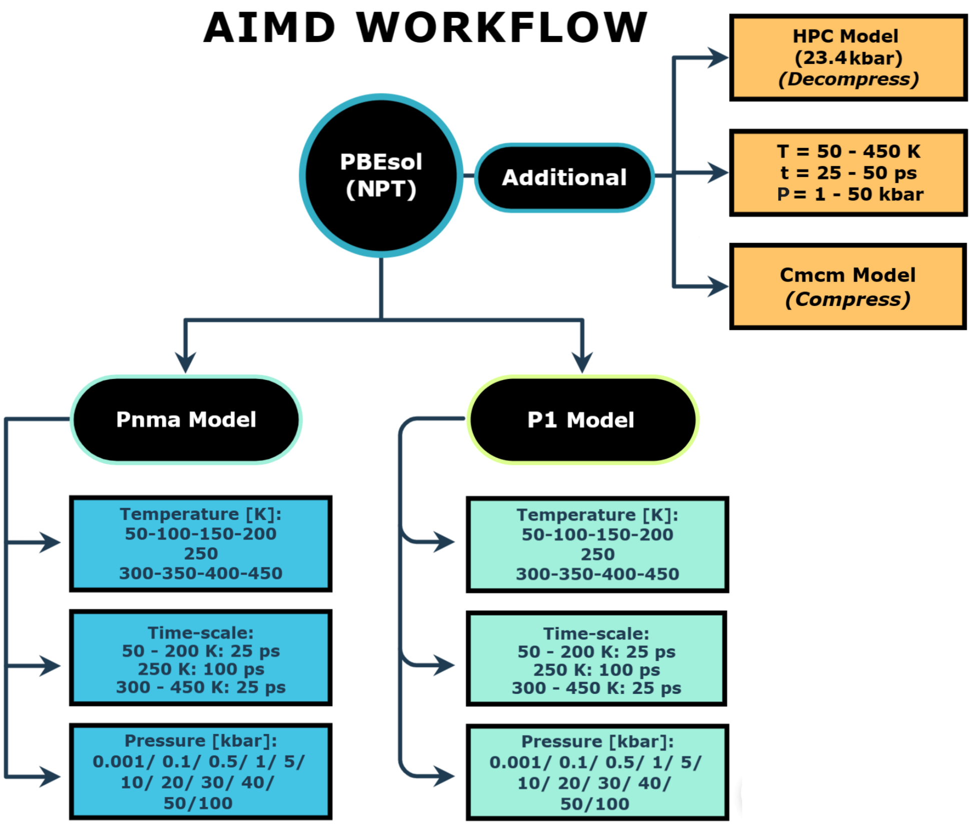


Figure S4: The computational workflow for the AIMD simulations adopted in the present work. Two reference structural models, namely, *Pnma* and *P*1, were subjected to extensive MD simulations, building the isobars across the range of 50 – 450 K at a given pressure, with the total time-scale of the simulations indicated in the figure. Starting from the base temperature (50 K), the final configuration was used to start the simulations at a higher temperature. Two additional models, namely *Cmcm* and *HPC* were subjected further simulations at selected thermodynamic conditions. These calculations were performed independently, *i.e.*, they do not account for the thermal history of a previous run.

# S3. Additional Computational Results

## S3.1. Vibrational Properties

In selected cases, the NPT simulations were followed by the set of fixed-volume 25 ps long AIMD runs in the canonical ensemble (NVT). These calculations employed the Canonical Sampling through Velocity Rescaling (CSVR) thermostat,[42] with the coupling constant of 10 fs and the temperature tolerance of 2.5 K for the global velocity rescaling. Finally, the AIMD

simulations were continued for 10 ps in the microcanonical (NVE) ensemble, allowing us to assess the semi-classical estimation of the thermodynamic properties. From these outputs, the Vibrational Density of States (VDoS; $g(E)$) was calculated by the Fourier transform of the mass-weighted Velocity AutoCorrelation Function (VACF).[43–45] For a given atom of mass $m_j$:

$$g(E) = \sum_{j}^{N} m_j \int_0^\infty \frac{< \nu(t)_j \nu(0)_j >}{< \nu(0)_j^2 >} exp\left(-i\frac{E}{\hbar}t\right) dt, \tag{1}$$

where $\nu(t)_i$ is the velocity at time $t$. Owing to the use of a relatively large integration step (1 fs), the frequency axis of the spectra has been corrected for the frequency shift of the Verlet integrator,[46] according to the expression:

$$\omega = \frac{\sqrt{2 - 2cos\left(\tilde{\omega}\Delta t\right)}}{\Delta t} \tag{2}$$

where $\Delta$t is the integrator time step and $\tilde{\omega}$ and $\omega$ stand for the observed and "true" frequency, respectively.

Fig. S5 provides a inspection of the low-energy part of the resulting mass-weighted VDoS for the *Pnma*, *P*1, and *Cmcm* models at selected temperatures and pressures. These modes can largely modulate the vibrational entropy. The partial contributions from the inorganic ($PbI_3^-$) and organic ($MA^+$) counterparts have been separated in the figure to highlight the origin of the vibrational contributions.

The VDoS were further used to approximate the vibrational entropy:[47]

$$S_{vib}^{VACF}\left(P,T\right) \approx -k_B \int_0^\infty \frac{g(E)}{\hbar} \left( ln\left(1 - e^{-E/k_BT}\right) + \frac{E}{k_B(1 - e^{E/k_BT})} \right) dE \tag{3}$$

where $k_B$ is the Boltzmann constant and $E$ is the energy of phonons.[48] Such an approach has been successfully applied to study the thermophysical properties of perovskites.[49]

The relative stability of the *Pnma*, *P*1, and *Cmcm* models is inspected by means of the

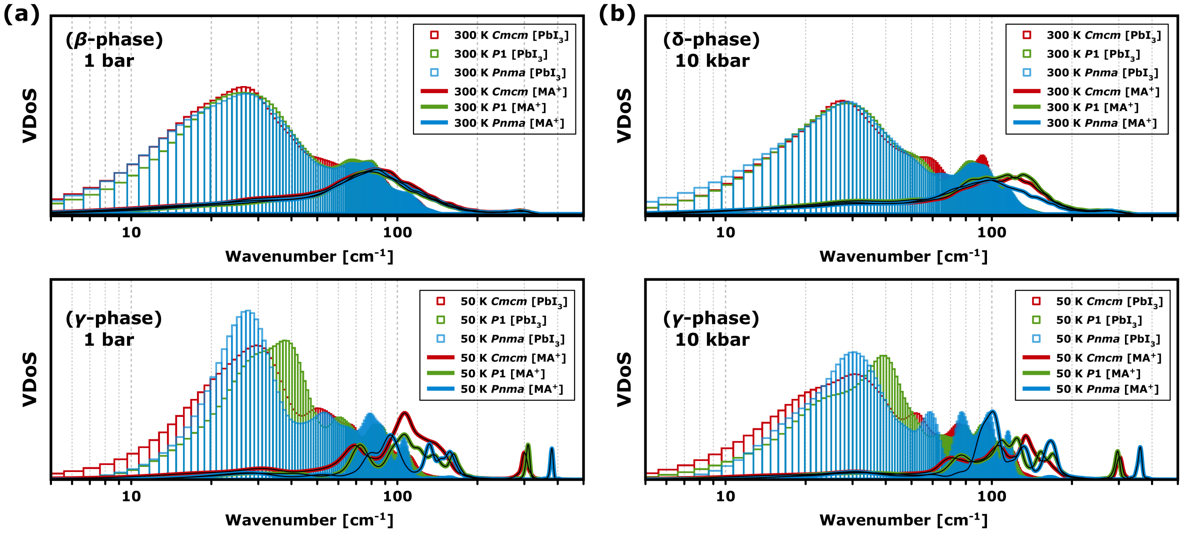


Figure S5: Partial VDoS in the low-frequency limit (< 500 cm$^{-1}$ in the log scale) decomposed into contributions from inorganic and organic parts. The results were obtained at 1 bar (a) and 10 kbar (b) at 50 K (bottom panels) and 300 K (upper panels), respectively.

Gibbs free energy, $G(P, T)$ – see Fig. S6. This thermodynamical potential was approximated as:

$$G(P, T) \approx U_{total} - T\Delta S_{vib}^{VACF} + PV \tag{4}$$

where $U_{total}$ is the ensemble average of internal energy, *i.e.*, potential energy plus the kinetic energy according to the NVE simulations and the PV term is obtained from the NPT simulations. As being handled by AIMD at the classical regime, the zero-point energy is not included.[50] However, supported by our previous findings,[37] we anticipate that in the present case these contributions are not decisive on the relative stability of the considered models.

Inspection of the left panel in Fig. S6 reveals a progressive increase of the potential energy as a function of temperature, taken directly as an average from NVE trajectories, accounting for the cell expansion beyond 0 K. This illustrates that the stabilization of the cation-disordered phases at elevated temperatures cannot be explained in terms of the 0 K potential energy considerations. On the other hand, the evolution of the Gibbs free energy estimated for each model under interest present in the right panel of Fig. S6 shows the opposite trend. In particular, at 1 bar one can note that the alternative models have already

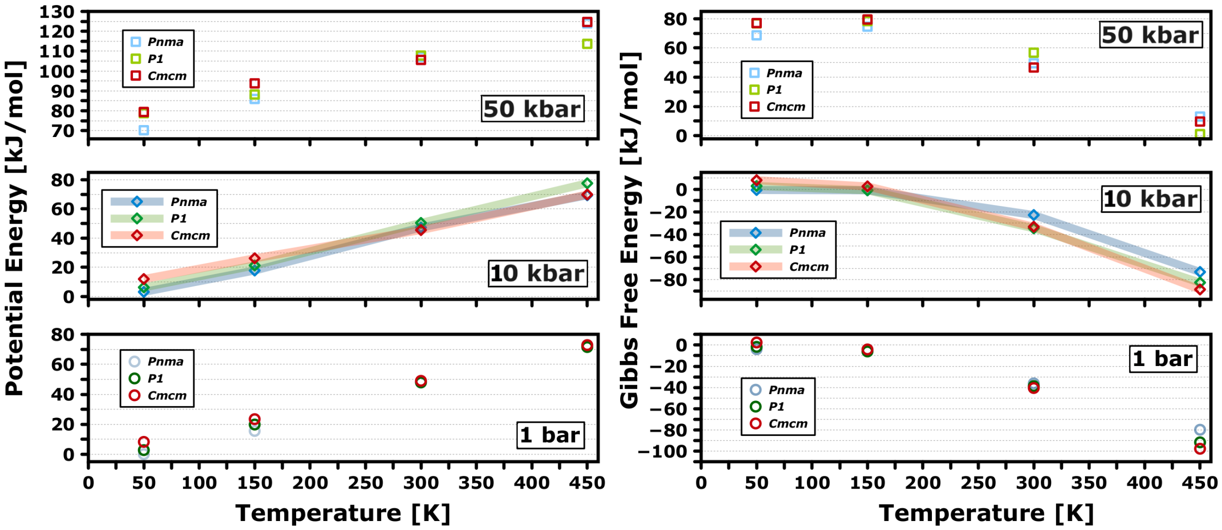


Figure S6: Potential energy (left panel) versus free energy (right panel) estimated from the variable-temperature NVE AIMD runs following the NPT simulations at three representative pressures.

become competitive at 150 K.

## S3.2. Evolution of the Perovskite Structure Across the P–T Space

We collected in Figure S7 the final snapshots from the NPT calculations across the $\gamma \rightarrow \beta$ transition. The resulting $\beta$ phase formed at ambient pressure is almost indistinguishable in the case of *Pnma* and *P*1 models.

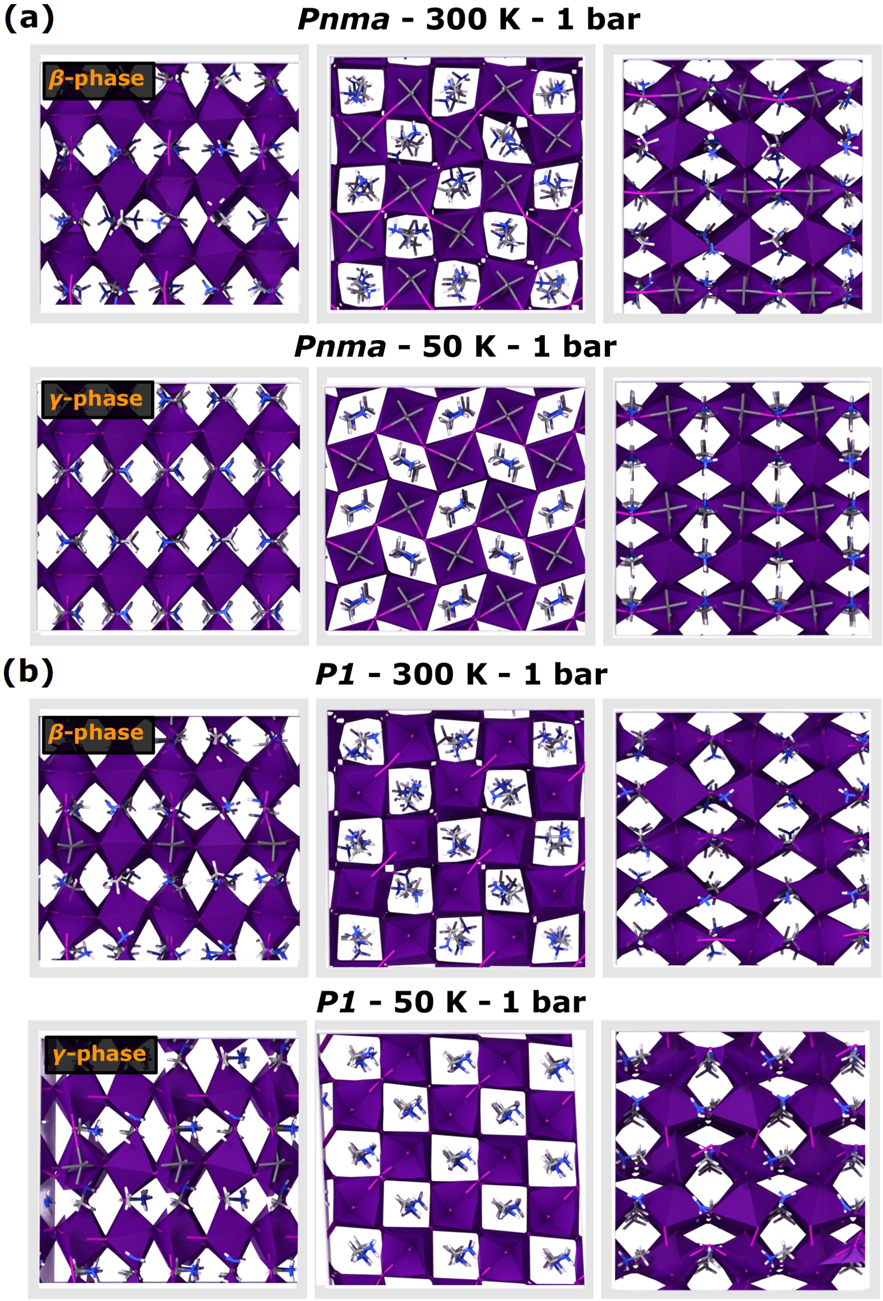


Figure S7: Final snapshots from the ambient pressure AIMD simulations at 50 and 300K (25 ps in the NPT ensemble), using two structural models: *Pnma* (a) and *P*1 (b).

Given that the pressurized *Pnma* model evolves to *P*1 at 300 K as deduced from the comparison of the average structures in Figure S8, the *P*1 model could be considered as an intermediate state model that triggers the transition to the $\delta$ phase.

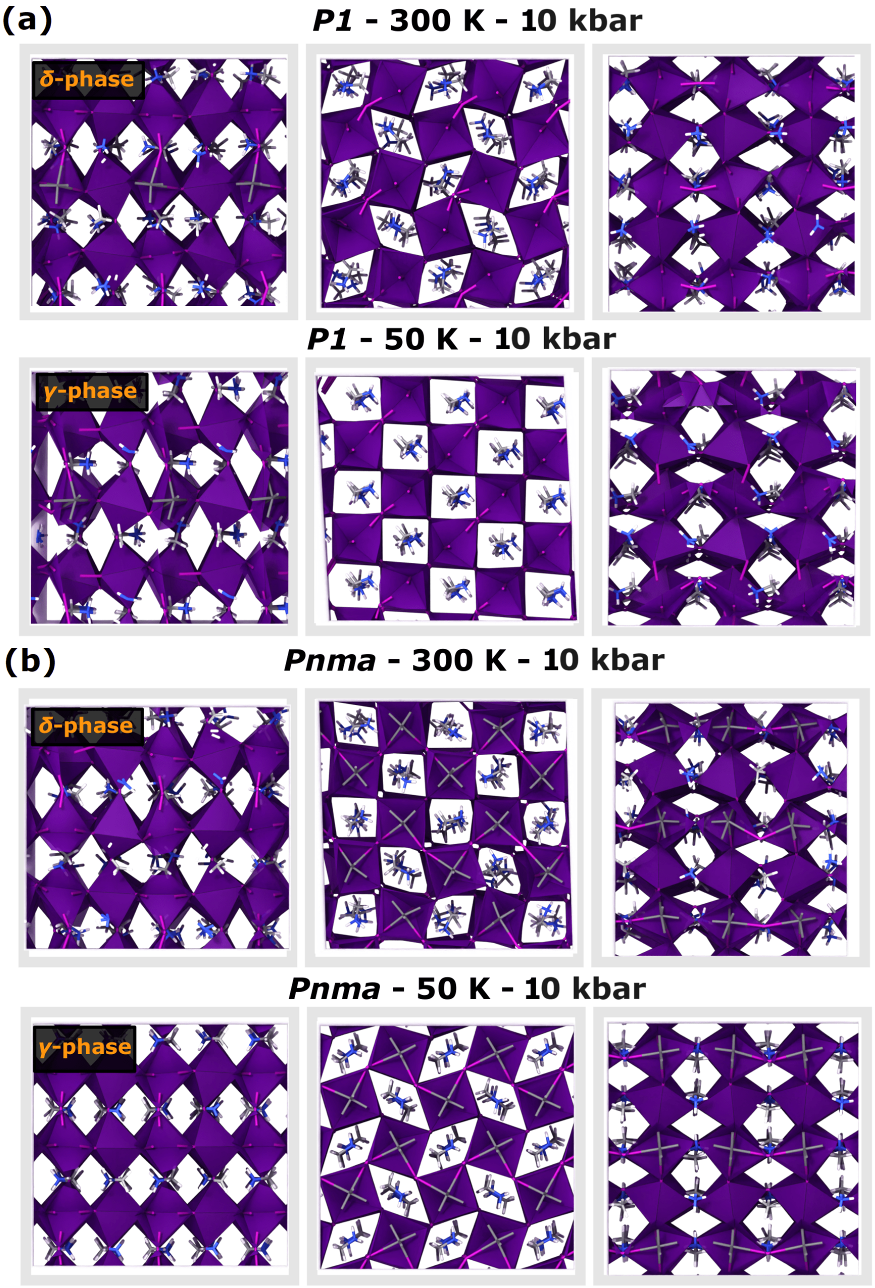


Figure S8: Final snapshots from the high-pressure (10 kbar) AIMD simulations at 50 and 300 K (25 ps in the NPT ensemble), using two structural models: *P*1 (a) and *Pnma* (b).

The postulated existence of the intermediate states poses a question on alternative mod-

els of the transition structures and calls for a more detailed inspection of the perovskite framework in terms of the octahedral tilting. The graph of maximal subgroups relating the space groups of the ideal cubic perovskite is presented in Fig. S9, indicating a possible scenario of the existence of the intermediate state described by the *Cmcm* symmetry. Such a scenario stays in line with our previous study on the low-temperature phase of $MAPbI_3$,[37] introducing the *Cmcm* model, which was found as the highest-energy stationary point on the potential energy landscape of the orientationally-ordered $\gamma$ phase.

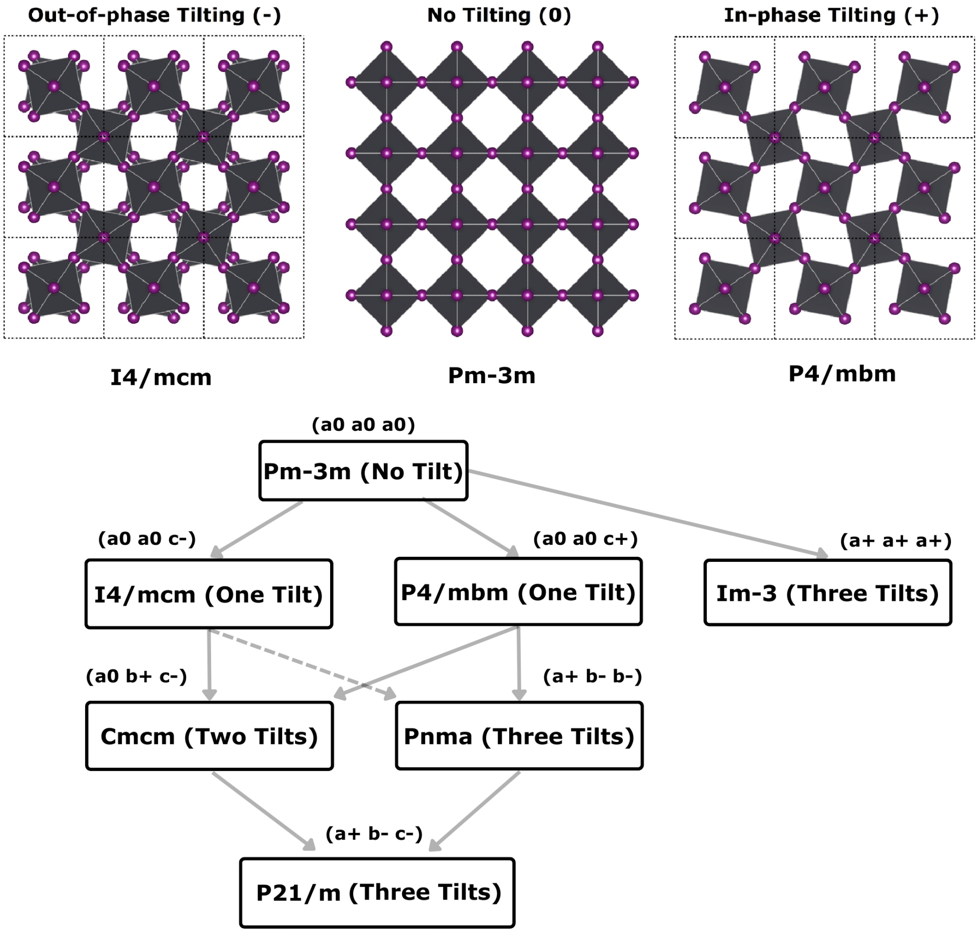


Figure S9: The Bärnighausen tree showing the group–subgroup relationships between the structures of $MAPbI_3$, noting the characteristic octahedral tilts of each structure using Glazer's notation. The dashed arrow indicates the first-order transition between the $I4/mcm$ and the *Pnma* structures.[51] The insets to the figures presented as the top panel illustrate the octahedral tilting in the two tetragonal hettotypes ($I4/mcm$ and $P4/mbm$, as projected along the long-molecular axis) compared to untilted cubic aristotype ($Pm\overline{3}m$).

To provide more detailed insights into the structure of largely similar models of the high-pressure $\delta$ phase, we refer to Alexandrov notation of possible octahedral tilts in distorted perovskite frameworks (see Fig. S10).

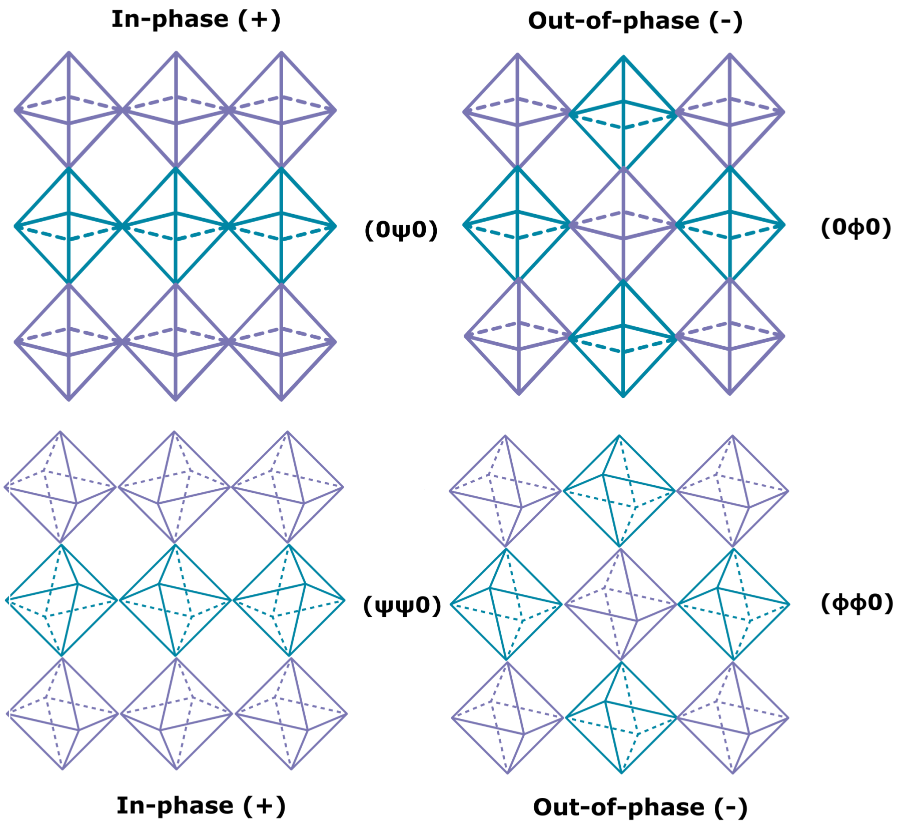


Figure S10: Octahedral tilting in distorted perovskites frameworks in Alexandrov notation as adopted from ref.[52] Rotations about the short cell axes ($a$ and $b$ by convention) can be either in-phase (+) or out-of-phase (-) and are represented by the Greek letters $\psi$ and $\phi$, respectively ($0\psi0$ and $0\phi0$). Simultaneous tilts around both $a$ and $b$ are also possible, as illustrated with the ($\psi\psi0$) and the ($\phi\phi0$) models.[52]

Such a classification has been used to characterize the resulting perovskite framework of each considered model as presented in Fig. S11 and referring to the average structures derived from AIMD simulations in the NVT ensemble following the NPT runs. In addition, the structure obtained upon decompression of the experimentally observed HPC model is given for comparison.

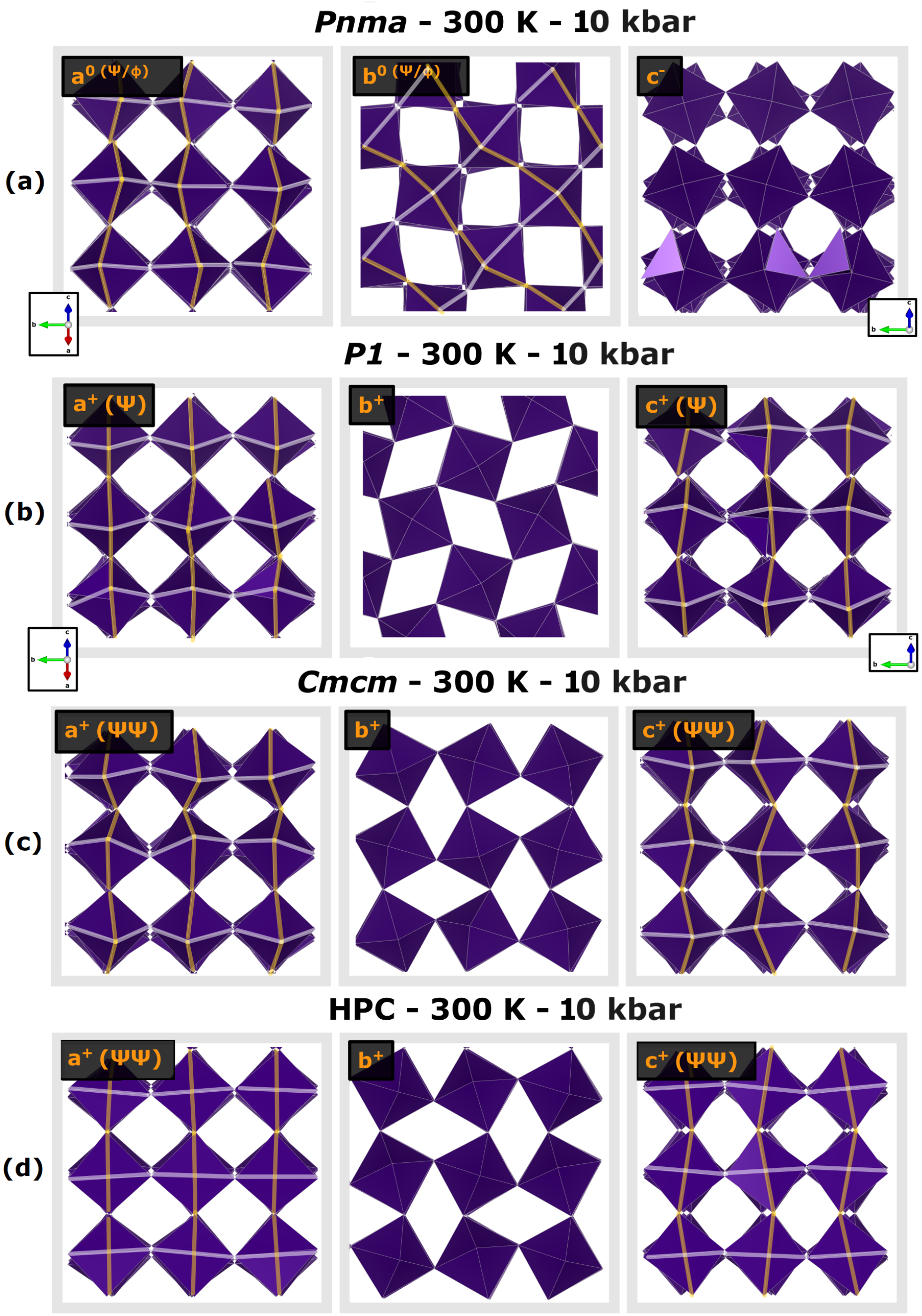


Figure S11: Average structures of $MAPbI_3$ at 10 kbar along with octahedra tiltings according to AIMD simulations at 300 K (15 ps in the NVT ensemble, following the NPT production runs). Four structural models considered: *Pnma* (a), *P*1 (b), *Cmcm* (c), and *HPC* (d). In each case, the methylammonium cations are omitted from display.

The continuously increasing pressure eventually leads to collapse of the perovskite structure (the $\epsilon$ phase, which has been illustrated in Fig. S12 by referring to the zero-tilt $\alpha$ phase.

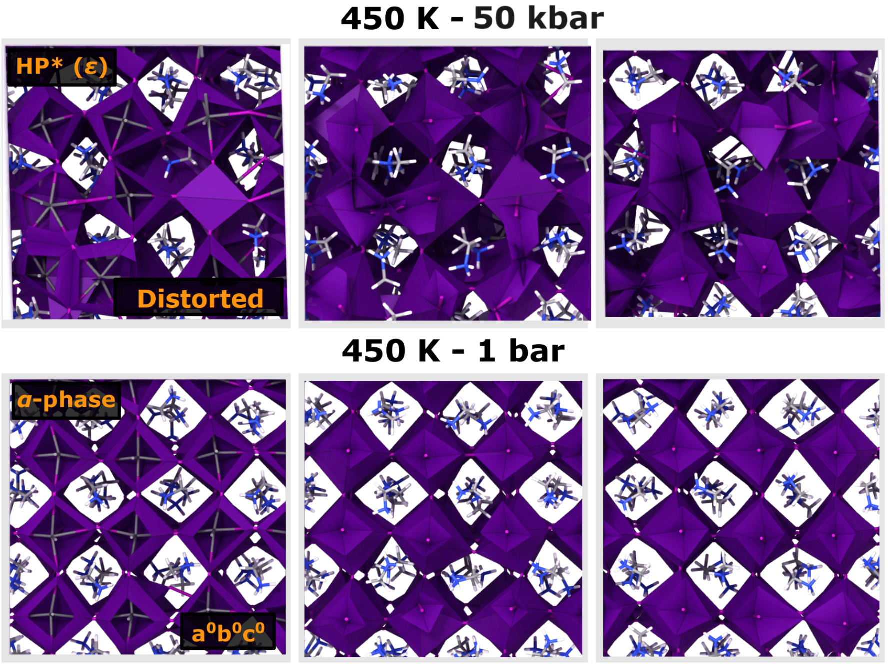


Figure S12: Final snapshots from 50 ps AIMD NPT simulations at 450 K and at ambient pressure (1 bar, bottom panel) and 50 kbar (upper panel).

A quantitative analysis of the pair correlation function, $g(\mathrm{r})$, present in Fig. S13 evinces a considerable change in the radial distribution functions at 50 kbar (r > 4 Å for each model), expressing the loss of the long-range ordering.

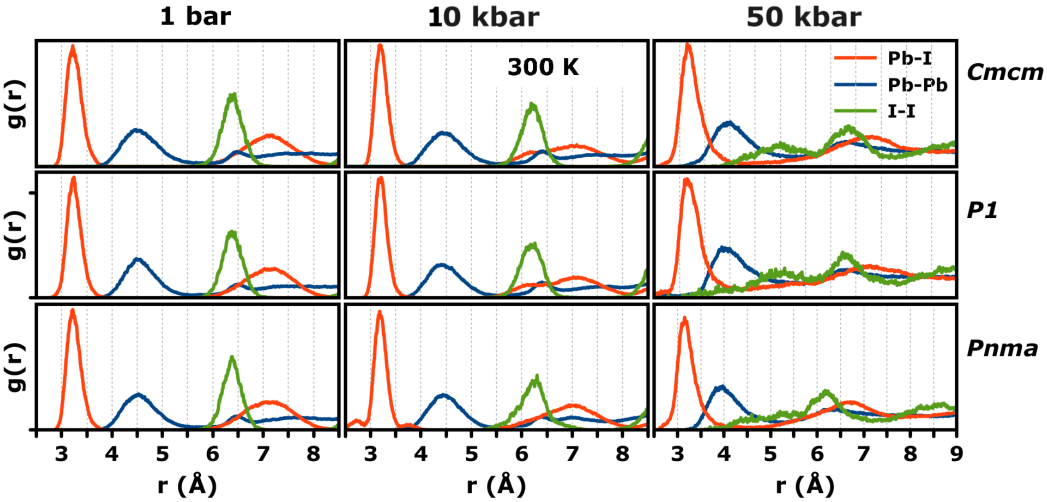


Figure S13: Pair correlation function, $g(\mathrm{r})$, of the inorganic components of $MAPbI_3$ derived from AIMD simulations (15 ps in the NVT ensemble, following the NPT production runs) for selected pressures at 300 K.

## S4. Quantitative Analysis of the Volumetric Properties

The evolution of the volumetric properties in $MAPbI_3$ observed experimentally was further analyzed quantitatively by means of the Clausius–Clapeyron method. This equation defines the tangent lines to the coexistence curve of two phases whatsoever in equilibrium. From the equality of chemical potentials, it follows that:

$$\frac{\Delta P}{\Delta T} = \frac{\Delta S}{\Delta V}. \tag{5}$$

The term on the left refers to the slope of the coexistence line between two phases in a pressure-temperature phase diagram. On the other hand, the volume per formula unit, $V_{FU}$, can be computed with the results extracted from the crystallographic refinements for the unit cell, leaving the change in entropy per formula unit as:

$$\Delta S_{FU} = \frac{\Delta P}{\Delta T} N_A \Delta V_{FU} \tag{6}$$

where $N_A$ is Avogadro's constant. The changes in entropy $\Delta S_{FU}$ derived from the recorded transitions in our OSIRIS runs are listed in Table S2 below. They are normalized to the gas constant $R$. Their standard uncertainties are computed from the fits for $\frac{\Delta P}{\Delta T}$ using the data reported by Gesi *et al.*[53]

Table S2: Changes in volume and entropy per formula-unit $\Delta S_{FU}$ across the P–T phase diagram of $MAPbI_3$ as calculated from equations 5 and 6 using the experimental data.

| Transition | Pressure | Temperature (K) | $\Delta V_{FU}$ (Å$^3$) | $\Delta S_{FU}$ ($R$) | $\Delta S_{FU}$ (J/kg·K) |
|---|---|---|---|---|---|
| $\gamma \to \beta$ | 1 bar | 165 ± 5 | 4.62 | 2.3 ± 0.2 | 30.8 ± 2.7 |
| $\gamma \to \beta$ | 0.5 kbar | 160 ± 20 | 4.73 | 2.4 ± 0.2 | 32.2 ± 2.7 |
| $\delta \to \beta$ | 1 kbar | 204 ± 15 | 5.95 | 0.7 ± 0.1 | 9.4 ± 1.3 |
| $\delta \to \beta$ | 2 kbar | 237 ± 17 | 5.49 | 0.7 ± 0.1 | 9.4 ± 1.3 |
| $\gamma \to \delta$ | 1 kbar | 160 ± 20 | -0.25 | 0.5 ± 0.3 | 6.7 ± 4.0 |
| $\gamma \to \delta$ | 2 kbar | 160 ± 20 | -0.38 | 0.8 ± 0.4 | 10.7 ± 5.4 |
| $\gamma \to \delta$ | 3 kbar | 160 ± 20 | -0.35 | 0.7 ± 0.4 | 9.4 ± 5.4 |
| $\gamma \to \delta$ | 4 kbar | 160 ± 20 | -0.36 | 0.7 ± 0.4 | 9.4 ± 5.4 |

The largest entropy changes characterize the ambient-pressure $\gamma \rightarrow \beta$ transition. The entropy change of $\Delta S = 2.3R$ stays in excellent agreement with the results by Onoda-Yamamuro and co-authors ($\Delta S$ = R$ln$9.8 = 2.28 $R$), reported based on the calorimetric measurements.[54] For the remaining transitions listed in Table S2, the changes of entropy are at the order of $0.7R$.

# References


(1) Beecher, A. N.; Semonin, O. E.; Skelton, J. M.; Frost, J. M.; Terban, M. W.; Zhai, H.; Alatas, A.; Owen, J. S.; Walsh, A.; Billinge, S. J. L. Direct Observation of Dynamic Symmetry Breaking above Room Temperature in Methylammonium Lead Iodide Perovskite. *ACS Energy Lett.* **2016**, *1*, 880–887.

(2) Ambrosio, F.; De Angelis, F.; Goñi, A. R. The Ferroelectric–Ferroelastic Debate about Metal Halide Perovskites. *J. Phys. Chem. Lett.* **2022**, *13*, 7731–7740.

(3) Breternitz, J. The "Ferros" of $MAPbI_3$: Ferroelectricity, Ferroelasticity and its Crystallographic Foundations in Hybrid Halide Perovskites. *Z. Kristallogr. Cryst. Mater.* **2022**, *237*, 135–140.

(4) Breternitz, J.; Lehmann, F.; Barnett, S. A.; Nowell, H.; Schorr, S. Role of the Iodide–Methylammonium Interaction in the Ferroelectricity of $CH_3NH_3PbI_3$. *Angew. Chem. Int. Ed.* **2019**, *59*, 424–428.

(5) Breternitz, J.; Tovar, M.; Schorr, S. Twinning in $CH_3NH_3PbI_3$ at Room Temperature Uncovered Through Laue Neutron Diffraction. *Sci. Rep.* **2020**, *10*, 16613–8.

(6) Liu, Y.; Collins, L.; Proksch, R.; Kim, S.; Watson, B. R.; Doughty, B.; Calhoun, T. R.; Ahmadi, M.; Ievlev, A. V.; Jesse, S.; et al. Chemical Nature of Ferroelastic Twin Domains in $CH_3NH_3PbI_3$ Perovskite. *Nat. Mater.* **2018**, *17*, 1013–1019.

(7) Li, B.; Kawakita, Y.; Liu, Y.; Wang, M.; Matsuura, M.; Shibata, K.; Ohira-Kawamura, S.; Yamada, T.; Lin, S.; Nakajima, K.; et al. Polar Rotor Scattering as Atomic-level Origin of Low Mobility and Thermal Conductivity of Perovskite $CH_3NH_3PbI_3$. *Nat. Commun.* **2017**, *8*, 16086–6.

(8) Whitfield, P. S.; Herron, N.; Guise, W. E.; Page, K.; Cheng, Y. Q.; Milas, I.; Crawford, M. K. Structures, Phase Transitions and Tricritical Behavior of the Hybrid Perovskite Methyl Ammonium Lead Iodide. *Sci. Rep.* **2016**, *6*, 35685–16.

(9) Fabini, D. H.; Hogan, T.; Evans, H. A.; Stoumpos, C. C.; Kanatzidis, M. G.; Seshadri, R. Dielectric and Thermodynamic Signatures of Low-Temperature Glassy Dynamics in the Hybrid Perovskites $CH_3NH_3PbI_3$ and $HC(NH_3)_3PbI_3$. *J. Phys. Chem. Lett.* **2016**, *7*, 376–381.

(10) Marín-Villa, P.; Arauzo, A.; Drużbicki, K.; Fernandez-Alonso, F. Unraveling the Ordered Phase of the Quintessential Hybrid Perovskite $MAPbI_3$ – Thermophysics to the Rescue. *J. Phys. Chem. Lett.* **2022**, *13*, 8422–8428.

(11) Drużbicki, K.; Pinna, R. S.; Rudić, S.; Jura, M.; Gorini, G.; Fernandez-Alonso, F. Unexpected Cation Dynamics in the Low-Temperature Phase of Methylammonium Lead Iodide: The Need for Improved Models. *J. Phys. Chem. Lett.* **2016**, *7*, 4701–4709.

(12) Drużbicki, K.; Lavén, R.; Armstrong, J.; Malavasi, L.; Fernandez-Alonso, F.; Karlsson, M. Cation Dynamics and Structural Stabilization in Formamidinium Lead Iodide Perovskites. *J. Phys. Chem. Lett.* **2021**, *12*, 3503–3508.

(13) Ou, T.; Yan, J.; Xiao, C.; Shen, W.; Liu, C.; Liu, X.; Han, Y.; Ma, Y.; Gao, C. Visible Light Response, Electrical Transport, and Amorphization in Compressed Organolead Iodine Perovskites. *Nanoscale* **2016**, *8*, 11426–11431.

(14) Capitani, F.; Marini, C.; Caramazza, S.; Postorino, P.; Garbarino, G.; Hanfland, M.; Pisanu, A.; Quadrelli, P.; Malavasi, L. High-pressure Behavior of Methylammonium Lead Iodide ($MAPbI_3$) Hybrid Perovskite. *J. Appl. Phys.* **2016**, *119*, 185901–6.

(15) Jaffe, A.; Lin, Y.; Beavers, C. M.; Voss, J.; Mao, W. L.; Karunadasa, H. I. High-Pressure Single-Crystal Structures of 3D Lead-Halide Hybrid Perovskites and Pressure Effects on their Electronic and Optical Properties. *ACS Cent. Sci.* **2016**, *2*, 201–209.

(16) Jiang, S.; Fang, Y.; Li, R.; Xiao, H.; Crowley, J.; Wang, C.; White, T. J.; Goddard, W. A.; Wang, Z.; Baikie, T.; *et al.* Pressure-dependent Polymorphism and Band-gap Tuning of Methylammonium Lead Iodide Perovskite. *Angew. Chem. Int. Ed.* **2016**, *55*, 6540–6544.

(17) Kong, L.; Liu, G.; Gong, J.; Hu, Q.; Schaller, R. D.; Dera, P.; Zhang, D.; Liu, Z.; Yang, W.; Zhu, K.; *et al.* Simultaneous Band-gap Narrowing and Carrier-lifetime Prolongation of Organic–inorganic Trihalide Perovskites. *Proc. Natl. Acad. Sci. U.S.A* **2016**, *113*, 8910–8915.

(18) Szafrański, M.; Katrusiak, A. Mechanism of Pressure-Induced Phase Transitions, Amorphization, and Absorption-Edge Shift in Photovoltaic Methylammonium Lead Iodide. *J. Phys. Chem. Lett.* **2016**, *7*, 3458–3466.

(19) Kong, L.; Gong, J.; Hu, Q.; Capitani, F.; Celeste, A.; Hattori, T.; Sano-Furukawa, A.; Li, N.; Yang, W.; Liu, G.; *et al.* Suppressed Lattice Disorder for Large Emission Enhancement and Structural Robustness in Hybrid Lead Iodide Perovskite Discovered by High-Pressure Isotope Effect. *Adv. Funct. Mater.* **2020**, *31*, 2009131–12.

(20) Demmel, F.; McPhail, D.; French, C.; Maxwell, D.; Harrison, S.; Boxall, J.; Rhodes, N.; Mukhopadhyay, S.; Silverwood, I.; Fernandez-Alonso, F.; *et al.* ToF-Backscattering Spectroscopy at the ISIS Facility: Status and Perspectives. *J. Phys. Conf. Ser.* **2018**, *1021*, 012027–5.

(21) Telling, M. T. F.; Campbell, S. I.; Engberg, D.; y Marero, D. M.; Andersen, K. H. Correction: Spectroscopic Characteristics of the OSIRIS Near-backscattering Crystal Analyser Spectrometer on the ISIS Pulsed Neutron Source. *Phys. Chem. Chem. Phys.* **2016**, *18*, 8243–8243.

(22) Demmel, F.; McPhail, D.; Crawford, J.; Maxwell, D.; Pokhilchuk, K.; Garcia-Sakai, V.; Mukhopadyay, S.; Telling, M.; Bermejo, F.; *et al.*, F. F.-A. Opening the Terahertz Window on the OSIRIS Spectrometer. *EPJ Web Conf.* **2015**, *83*, 03003–4.

(23) Telling, M. T. F.; Andersen, K. H. Spectroscopic Characteristics of the OSIRIS Near-backscattering Crystal Analyser Spectrometer on the ISIS Pulsed Neutron Source. *Phys. Chem. Chem. Phys.* **2005**, *7*, 1255–1261.

(24) Demmel, F.; Perrichon, A.; McPhail, D.; Luna Dapica, P.; Webb, N.; Cook, A.; Schooneveld, E.; Boxall, J.; Rhodes, N.; Fernandez-Alonso, F.; *et al.* Silver Jubilee for the OSIRIS Spectrometer: Achievements and Outlook. *EPJ Web Conf.* **2023**, *286*, 03005–6.

(25) Arnold, O.; Bilheux, J.; Borreguero, J.; Buts, A.; Campbell, S.; Chapon, L.; Doucet, M.; Draper, N.; Leal, R. F.; Gigg, M.; *et al.* Mantid—Data Analysis and Visualization Package for Neutron Scattering and $\nu$SR experiments. *Nucl. Instrum. Methods Phys. Res.* **2014**, *764*, 156–166.

(26) Toby, B. H.; Von Dreele, R. B. GSAS-II: The Genesis of a Modern Open-source All Purpose Crystallography Software Package. *J. Appl. Crystallogr.* **2013**, *46*, 544–549.

(27) Lehmann, F.; Franz, A.; Többens, D. M.; Levcenco, S.; Unold, T.; Taubert, A.; Schorr, S. The Phase Diagram of a Mixed Halide (Br, I) Hybrid Perovskite Obtained by Synchrotron X-ray Diffraction. *RSC Adv.* **2019**, *9*, 11151–11159.

(28) Lotti, P.; Milani, S.; Merlini, M.; Joseph, B.; Alabarse, F.; Lausi, A. Single-crystal

Diffraction at the High-pressure Indo-Italian Beamline Xpress at Elettra, Trieste. *J. Synchrotron Radiat.* **2020**, *27*, 222–229.

(29) Datchi, F.; Dewaele, A.; Loubeyre, P.; Letoullec, R.; Le Godec, Y.; Canny, B. Optical Pressure Sensors for High-pressure-High-temperature Studies in a Diamond Anvil Cell. *High Press. Res.* **2007**, *27*, 447–463.

(30) Kühne, T. D.; Iannuzzi, M.; Ben, M. D.; Rybkin, V. V.; Seewald, P.; Stein, F.; Laino, T.; Khaliullin, R. Z.; Schütt, O.; Schiffmann, F.; *et al.* CP2K: An Electronic Structure and Molecular Dynamics Software Package - Quickstep: Efficient and Accurate Electronic Structure Calculations. *J. Chem. Phys.* **2020**, *152*, 194103–47.

(31) Hutter, J.; Iannuzzi, M.; Schiffmann, F.; VandeVondele, J. CP2K: Atomistic Simulations of Condensed Matter Systems. *Wiley Interdiscip. Rev. Comput. Mol. Sci.* **2013**, *4*, 15–25.

(32) Carignano, M. A.; Kachmar, A.; Hutter, J. Thermal Effects on $CH_3NH_3PbI_3$ Perovskite from Ab Initio Molecular Dynamics Simulations. *J. Phys. Chem. C* **2015**, *119*, 8991–8997.

(33) Carignano, M. A.; Aravindh, S. A.; Roqan, I. S.; Even, J.; Katan, C. Critical Fluctuations and Anharmonicity in Lead Iodide Perovskites from Molecular Dynamics Supercell Simulations. *J. Phys. Chem. C* **2017**, *121*, 20729–20738.

(34) Kaiser, W.; Carignano, M.; Alothman, A. A.; Mosconi, E.; Kachmar, A.; Goddard, W. A.; Angelis, F. D. First-Principles Molecular Dynamics in Metal-Halide Perovskites: Contrasting Generalized Gradient Approximation and Hybrid Functionals. *J. Phys. Chem. Lett.* **2021**, *12*, 11886–11893.

(35) Ambrosio, F.; Meggiolaro, D.; Mosconi, E.; Angelis, F. D. Charge Localization, Stabilization, and Hopping in Lead Halide Perovskites: Competition between Polaron Stabilization and Cation Disorder. *ACS Energy Lett.* **2019**, *4*, 2013–2020.

(36) Perdew, J. P.; Ruzsinszky, A.; Csonka, G. I.; Vydrov, O. A.; Scuseria, G. E.; Constantin, L. A.; Zhou, X.; Burke, K. Restoring the Density-Gradient Expansion for Exchange in Solids and Surfaces. *Phys. Rev. Lett.* **2008**, *100*, 136406–4.

(37) Drużbicki, K.; Gila-Herranz, P.; Marin-Villa, P.; Gaboardi, M.; Armstrong, J.; Fernandez-Alonso, F. Cation Dynamics as Structure Explorer in Hybrid Perovskites The Case of $MAPbI_3$. *Cryst. Growth Des.* **2023**, *24*, 391–404.

(38) Lahnsteiner, J.; Kresse, G.; Kumar, A.; Sarma, D. D.; Franchini, C.; Bokdam, M. Room-temperature Dynamic Correlation Between Methylammonium Molecules in Lead-iodine Based Perovskites: an Ab Initio Molecular Dynamics Perspective. *Phys. Rev. B* **2016**, *94*, 214114–10.

(39) Bokdam, M.; Lahnsteiner, J.; Ramberger, B.; Schäfer, T.; Kresse, G. Assessing Density Functionals Using Many Body Theory for Hybrid Perovskites. *Phys. Rev. Lett.* **2017**, *119*, 145501–5.

(40) Lahnsteiner, J.; Kresse, G.; Heinen, J.; Bokdam, M. Finite-temperature Structure of the $MAPbI_3$ Perovskite: Comparing Density Functional Approximations and Force Fields to Experiment. *Phys. Rev. Mater.* **2018**, *2*, 073604–14.

(41) Martyna, G. J.; Tuckerman, M. E.; Tobias, D. J.; Klein, M. L. Explicit Reversible Integrators for Extended Systems Dynamics. *Mol. Phys.* **1996**, *87*, 1117–1157.

(42) Bussi, G.; Donadio, D.; Parrinello, M. Canonical Sampling Through Velocity Rescaling. *J. Chem. Phys.* **2007**, *126*, 014101–7.

(43) Thomas, M.; Brehm, M.; Fligg, R.; Vöhringer, P.; Kirchner, B. Computing Vibrational Spectra from *Ab Initio* Molecular Dynamics. *Phys. Chem. Chem. Phys.* **2013**, *15*, 6608–6622.

(44) Brehm, M.; Kirchner, B. TRAVIS - A Free Analyzer and Visualizer for Monte Carlo and Molecular Dynamics Trajectories. *J. Chem. Inf. Model.* **2011**, *51*, 2007–2023.

(45) Brehm, M.; Thomas, M.; Gehrke, S.; Kirchner, B. TRAVIS—A Free Analyzer for Trajectories from Molecular Simulation. *J. Chem. Phys.* **2020**, *152*, 164105–20.

(46) Verlet, L. Computer "Experiments" on Classical Fluids. I. Thermodynamical Properties of Lennard-Jones Molecules. *Phys. Rev.* **1967**, *159*, 98–103.

(47) Escorihuela–Sayalero, C.; Pardo, L. C.; Romanini, M.; Obrecht, N.; Loehlé, S.; Lloveras, P.; Tamarit, J.; Cazorla, C. Prediction and Understanding of Barocaloric Effects in Orientationally Disordered Materials from Molecular Dynamics Simulations. *NPJ Comput. Mater.* **2024**, *10*, 1–10.

(48) Korotaev, P.; Belov, M.; Yanilkin, A. Reproducibility of Vibrational Free Energy by Different Methods. *Comp. Mat. Sci.* **2018**, *150*, 47–53.

(49) Braeckevelt, T.; Goeminne, R.; Vandenhaute, S.; Borgmans, S.; Verstraelen, T.; Steele, J. A.; Roeffaers, M. B. J.; Hofkens, J.; Rogge, S. M. J.; Van Speybroeck, V. Accurately Determining the Phase Transition Temperature of $CsPbI_3$ via Random-Phase Approximation Calculations and Phase-Transferable Machine Learning Potentials. *Chem. Mater.* **2022**, *34*, 8561–8576.

(50) Zhang, Z.; Zhang, D.-B.; Sun, T.; Wentzcovitch, R. M. The Phonon Quasiparticle Approach for Anharmonic Properties of Solids. *J. Phys. Conf. Ser.* **2022**, *2207*, 012042–12.

(51) Even, J.; Carignano, M.; Katan, C. Molecular Disorder and Translation/rotation Coupling in the Plastic Crystal Phase of Hybrid Perovskites. *Nanoscale* **2016**, *8*, 6222–6236.

(52) Liu, T.; Holzapfel, N. P.; Woodward, P. M. Understanding Structural Distortions in

Hybrid Layered Perovskites with the n = 1 Ruddlesden-Popper structure. *IUCrJ* **2023**, *10*, 385–396.

(53) Gesi, K. Effect of Hydrostatic Pressure on the Structural Phase Transitions in $CH_3NH_3PbX_3$ (X = Cl, Br, I). *Ferroelectrics* **1997**, *203*, 249–268.

(54) Onoda-Yamamuro, N.; Matsuo, T.; Suga, H. Calorimetric and IR Spectroscopic Studies of Phase Transitions in Methylammonium Trihalogenoplumbates (II). *J. Phys. Chem. Solids* **1990**, *51*, 1383–1395.
